# Clinical associations and characteristics of the polyspecific intrathecal immune response in elderly patients with non-multiple sclerosis chronic autoimmune-inflammatory neurological diseases – a retrospective cross-sectional study

**DOI:** 10.3389/fneur.2023.1193015

**Published:** 2023-06-15

**Authors:** Felix Brauchle, Daniel Rapp, Makbule Senel, André Huss, Jens Dreyhaupt, Veronika Klose, Marie Süße, Klarissa Hanja Stürner, Frank Leypoldt, Hayrettin Tumani, Jan Lewerenz

**Affiliations:** ^1^Department of Neurology, Ulm University, Ulm, Germany; ^2^Institute of Epidemiology and Medical Biometry, Ulm University, Ulm, Germany; ^3^German Center for Neurodegenerative Diseases (DNZE), Campus Ulm, Ulm, Germany; ^4^Department of Neurology, University Medicine Greifswald, Greifswald, Germany; ^5^Department of Neurology, University Hospital Schleswig-Holstein, Campus Kiel, Kiel, Germany; ^6^Neuroimmunology, Institute of Clinical Chemistry, University Hospital Schleswig-Holstein, Campus Kiel, Kiel, Germany

**Keywords:** polyspecific intrathecal immune response, MRZ reaction, multiple sclerosis, neuroinflammation, autoimmue disease

## Abstract

**Introduction:**

The polyspecific intrathecal immune response (PSIIR), aka MRZ reaction (*M* = measles, *R* = rubella, *Z* = zoster, optionally Herpes simplex virus, HSV) is defined as intrathecal immunoglobulin synthesis (IIS) for two or more unrelated viruses. Although an established cerebrospinal fluid (CSF) biomarker for multiple sclerosis (MS), a chronic autoimmune-inflammatory neurological disease (CAIND) of the central nervous system (CNS) usually starting in young adulthood, the full spectrum of CAINDs with a positive PSIIR remains ill defined.

**Methods:**

In this retrospective, cross-sectional study, patients with CSF-positive oligoclonal bands (OCB) and - to enrich for non-MS diagnoses - aged ≥50 years were enrolled.

**Results:**

Of 415 with PSIIR testing results (MRZ, HSV optional), 76 were PSIIR-positive. Of these, 25 (33%) did not meet the diagnostic criteria for MS spectrum diseases (MS-S) comprising clinically or radiologically isolated syndrome (CIS/RIS) or MS. PSIIR-positive non-MS-S phenotypes were heterogenous with CNS, peripheral nerve and motor neuron involvement and often defied unequivocal diagnostic classification. A rating by neuroimmunology experts suggested non-MS CAINDs in 16/25 (64%). Long-term follow-up available in 13 always showed a chronically progressive course. Four of five responded to immunotherapy. Compared to MS-S patients, non-MS CAIND patients showed less frequent CNS regions with demyelination (25% vs. 75%) and quantitative IgG IIS (31% vs. 81%). MRZ-specific IIS did not differ between both groups, while additional HSV-specific IIS was characteristic for non-MS CAIND patients.

**Discussion:**

In conclusion, PSIIR positivity occurs frequently in non-MS-S patients ≥50 years. Although sometimes apparently coincidental, the PSIIR seems to represent a suitable biomarker for previously unnoticed chronic neurologic autoimmunities, which require further characterization.

## Introduction

Cerebrospinal fluid (CSF) analysis is an important part of the diagnostic work-up of suspected neurologic autoimmunities. Multiple sclerosis (MS) is the most common chronic autoimmune-inflammatory neurological disease (CAIND), which typically manifests in young adulthood (1). Late-onset MS (LOMS) starting at an age ≥ 50 years is rare (2). More than 95% of MS patients show intrathecal immunoglobulin synthesis (IIS) of IgG detectable as oligoclonal bands (OCB) (3–5). OCB, however, occur in many inflammatory neurological diseases, infectious or autoimmune (4, 6, 7), in up to 5% of healthy subjects (8, 9) and – by coincidence - in non-inflammatory neurological diseases (10).

The polyspecific intrathecal humoral immune response (PSIIR) is a composite antibody biomarker usually measured as MRZ reaction (*M* = measles, *R* = rubella, *Z* = zoster) (11). However, the PSIIR can comprise other viruses, e.g., Herpes simplex virus (HSV) (7). Polyspecificity is defined as pathogen-specific IIS for two or more unrelated infectious agents with high seroprevalence (12, 13), none of which should elevated due to a previous or current infection of the nervous system with the respective virus. Pathogen-specific IIS is usually measured as the pathogen-specific CSF/serum antibody index (AI) (14, 15). Unspecific recruitment of circulating memory *B* cells to the central nervous system (CNS) is thought to underlie the PSIIR (16, 17). Within the CNS, the MS-type chronic inflammatory response provides niches for their antigen-independent proliferation and subsequent antibody production (16, 17).

The PSIIR is positive in ~70% of MS patients (11, 18). Its specificity for MS reportedly exceeds 90% (11, 18). CAINDs as part of several well-defined connective tissue diseases (CTD), e.g., systemic lupus erythematosus (SLE) (19, 20), Sjögren’s syndrome and granulomatosis with polyangiitis (20), may be PSIIR-positive but at much lower frequencies when compared to MS (15–20%) (11). In other inflammatory neurological diseases, a PSIIR is thought to be exceedingly rare (<4%), even more so in non-inflammatory neurological disease (<0.5%) (11) and in healthy volunteers (0/99) (9). This makes the PSIIR a highly interesting biomarker for CAINDs.

Surprisingly, no systematic approaches to identify PSIIR-positive non-MS CAINDs have been published. In general, reports of non-MS PSIIR-positive patients are compilations of reported cases or small case series (11) or report the results of testing for the PSIIR in pre-defined CTDs with nervous system involvement (19). We reported recently that PSIIR positivity occurs in more than one third of cases suffering from autoimmune encephalitis with NMDA receptor antibodies (NMDAR-E) (21, 22), a surprising finding as unlike MS this disease rather subacute, not chronic. However, this finding indicates that additional PSIIR-positive autoimmune-inflammatory neurologic diseases remain to be discovered.

In a non-hypothesis driven approach, we performed a retrospective, monocentric, cross-sectional study using a large cohort of OCB-positive patients tested consecutively for the PSIIR (MRZ, HSV AI optional) at the time of diagnostic work-up to identify new or known non-MS PSIIR-positive CAINDs. To increase the likelihood of non-MS diagnoses, the minimum age for inclusion was set to 50 years.

## Materials and methods

### Patient identification

The database of the Laboratory for CSF Diagnostics and Clinical Neurochemistry of the Department of Neurology, Ulm University, was retrospectively screened for in-patient CSF results from June 1^st^, 2007 to May 31st, 2017 fulfilling the following inclusion criteria: patient age ≥ 50 years, OCB-positive, no relevant blood contamination (CSF erythrocyte count <500/μL), AI measurement for measles, rubella and VZV, facultatively also for HSV, performed at the time of diagnostic work-up. The AIs were either ordered by the treating physician or performed by the CSF laboratory due an obviously chronic-inflammatory CSF pattern. PSIIR was rated positive when AIs for at least two unrelated viruses were elevated (14) (>1.4, measles + rubella, measles + VZV/HSV, rubella + VZV/HSV, measles + rubella + VZV/HSV). Upon seronegativity for a pathogen or when the CSF titer was below the lower limit of quantification (LLQ), the respective AI cannot be calculated. Cases were only rated definitely PSIIR-negative when 2 of 3 AIs (or 3 of 4 AIs when HSV AI was tested) could be calculated and were in the normal range (0.6–1.4).

For PSIIR-positive cases, the patients’ history, findings upon neurological examination, results of additional laboratory tests as well as those of spinal and cranial MRI were extracted from the patients’ charts. Based on the medical records and MRI re-analysis (see below), all PSIIR-positive patients were tested whether they fulfilled the current diagnostic criteria for MS spectrum (MS-S) diseases, including relapsing–remitting and primary progressive MS (RRMS/PPMS) (23), clinically isolated syndrome (CIS) (24) or radiologically isolated syndrome (RIS) (25). Depending on data availability, systemic autoimmune diseases were confirmed according to the most recent diagnostic criteria (26–29).

### Cerebrospinal fluid analysis

Cerebrospinal fluid leukocyte count was performed manually using a Fuchs-Rosenthal hematocytometer with the upper limit of normal (ULN) of 4 leukocytes/μL. OCB were visualized using isoelectric focusing as described (30). Pathogen-specific IgG CSF/serum ratios (Q_spec_) were quantified by enzyme-linked immunosorbent assay (Virion/Serion, Würzburg, Germany) as described (31). CSF lactate was measured photometrically using AU400/AU680 Clinical Chemistry Analyzers (Olympus/Beckman Coulter, Krefeld, Germany) with an age-dependent normal range of 1.7–2.6 mmoL/L. Albumin, IgG, IgA and IgM in CSF and serum were quantified using a BN Prospec nephelometer (Siemens, Munich, Germany). For the CSF/serum albumin ratio (Q_Alb_), the age-dependent ULN (Q_Alb_lim_) was calculated as 4 + (age (years)/15) × 10^−3^ (32)_._ The age-normalized Q_Alb_ (Q_Alb_Age_) was calculated as Q_Alb_/Q_Alb_lim_ (ULN 1.0). Quantitative IgG, IgA and IgM IIS is defined as definitely higher concentration of the respective immunoglobulin in the CSF from what is expected in comparison with its serum concentration and the Q_Alb_. Quantitative IIS was assessed according to the Reiber formulas and positive when Q_IgG/A/M_ > ULN (Q_lim_high_) (7). Pathogen-specific AIs were calculated by dividing the pathogen-specific CSF/serum IgG (Q_spec_) by Q_IgG_ or Q_lim_high_, whichever was lower (14) (ULN = 1.4). Z score conversion of Q_IgG_ (zQ_IgG_) and Q_spec_ (zQ_spec_) was performed as described in detail in the Supplementary Methods section. We categorized IIS as possible when the zQ_IgG_ was >1 and ≤2, probable when >2 and ≤3 and definite when >3. To test whether pathogen-specific IgG IS exceeds total IgG IS, zQ_IgG_ was subtracted from zQ_spec_ (ΔzQ_spec_/zQ_IgG_).

### MRI analysis

Cranial and spinal MRIs were performed on a 1.5 tesla MRI (Symphony, Siemens, Erlangen, Germany) during diagnostic work-up. MRIs of PSIIR-positive patients were re-analyzed for periventricular, juxtacortical/cortical, infratentorial and spinal lesions (≥3 mm) suggestive for demyelination to test the current MRI criteria for dissemination in space and time. The lesion load was calculated as the percentage of regions with lesions of all regions with images available, as not all patients had spinal MRIs (Supplementary Table 1).

### Expert rating

To objectify how convincingly the clinical data suggested a non-MS CAIND in the non-MS-S PSIIR-positive patients, we performed a survey with four experts in clinical neuroimmunology (FL, KHS, MSe, and HT) using detailed case descriptions (see Appendix 1/2). Each case was rated by each expert independently for the agreement with the statements that a. the patient does not suffer from MS (no MS score) and b. the patient suffers from a CAIND (CAIND score) using Likert scales (0 = totally disagree, 25 = rather disagree, 50 = undecided, 75 = rather agree, 100 = totally agree, for details see Supplementary Methods).

### Statistical analysis

Statistical analysis was performed using GraphPad Prism (version 8.4.2; GraphPad Software Inc., San Diego, United States). For continuous variables, differences between two groups were analyzed by the Mann–Whitney U, between two or more groups by Kruskal-Wallis followed Bonferroni-corrected Dunn’s post-tests. As global test for categorial data, Fisher’s exact tests were used for 4-field table comparisons. For more than four fields, the Chi-square tests were applied after testing whether the requirements for this test were met. For multiple Fisher’s exact tests performed as post-hoc tests for a Chi-square test, the *p* values were Bonferroni-adjusted. As a retrospective analysis was performed, the results must be categorized as hypothesis generating.

## Results

### Study recruitment

**Figure 1:**
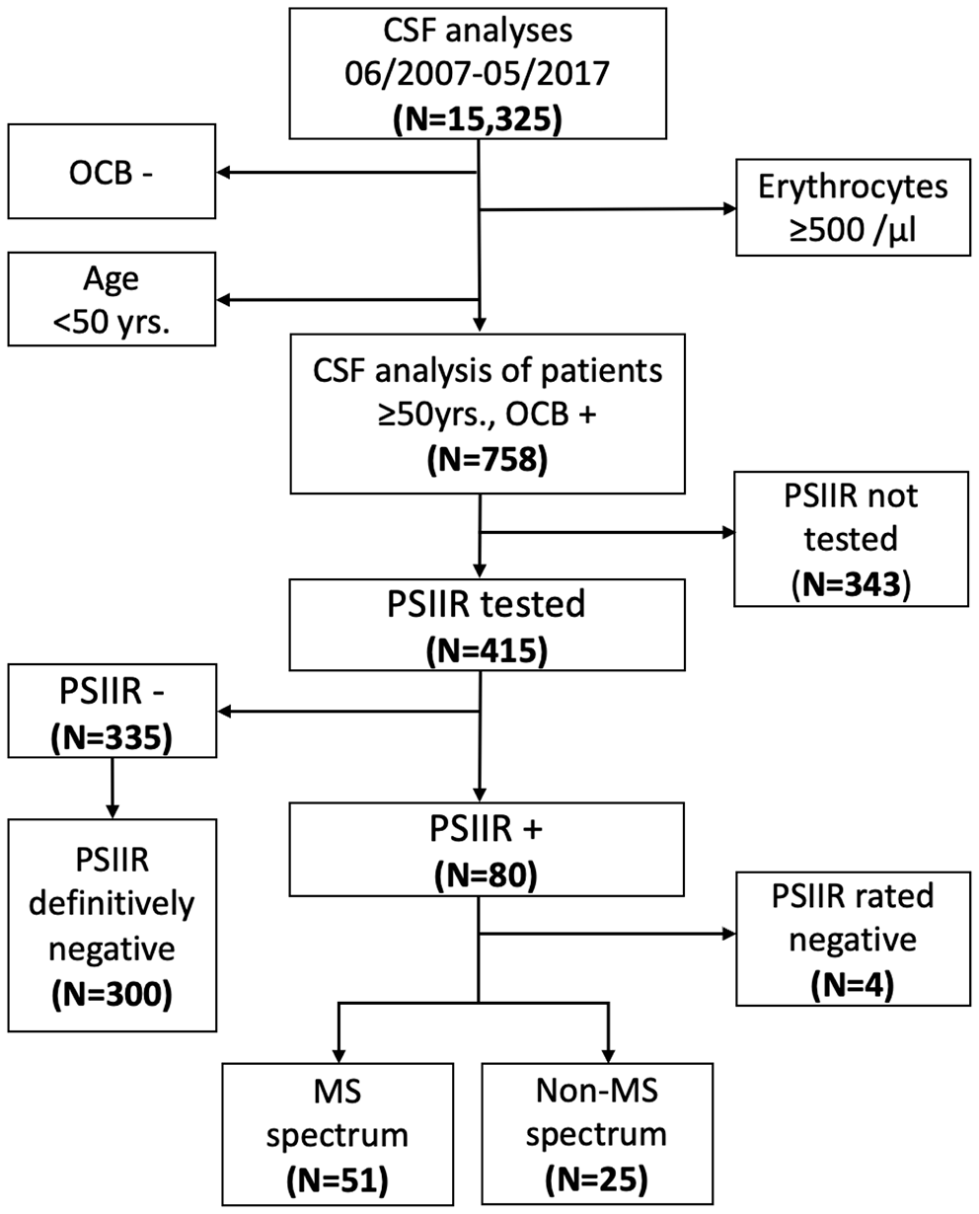
Flowchart of the study profile. CSF: cerebrospinal fluid, OCB−/+: oligoclonal bands negative/positive, PSIIR−/+: polyspecific intrathecal immune response negative/positive, MS: multiple sclerosis. PSIIR definitely negative: patients seronegative for only one of the pathogens tested and all other CSF/serum antibody indices (AI) normal or no seronegativity for any pathogen tested when one AI was elevated. PSIIR reaction rated negative: the PSIIR reaction was rated negative due to infection-related AI elevation or plasmapheresis-induced CSF/plasma disequilibrium.

Among the 15,325 subsequent CSF analyses, 758 patients were ≥ 50 years old and OCB-positive (Figure 1). Of those, 415 patients (55%) had been tested for the PSIIR comprising AIs for measles, rubella and VZV at the time of diagnostic work-up. In 224/415 (54%), these analyses were complemented by a HSV AI. In 27 (7%), 54 (13%) and eight (2%) of the 415 patients, measles, rubella or VZV antibodies, respectively, were either negative or CSF titers were too low for AI calculation. This was the case in 20/224 (9%) tested for HSV. Overall, in 161/415 patients (39%) all four AIs were available, in 216 (52%) three AIs, in 30 (7%) only two, and in 8 (2%) only one. Two or more AIs were elevated in in 80 (19%). However, in three patients one of the two elevated AIs was elevated due to exposure with the respective pathogen: one each had previous measles meningitis, HSV encephalitis and VZV reactivation. A fourth patient showed multiple elevated AIs following plasma exchange. This elevation was most likely caused by a persisting CSF/plasma disequilibrium with blood IgG still decreased compared to CSF at the time of lumbar puncture. After exclusion of these four (Figure 1), 76 PSIIR-positive patients remained. With too many AIs missing due to seronegativity or antibody levels below the LLQ in CSF, 35 PSIIR-negative patients were excluded, as it could not ascertained whether the underlying pathophysiology would have let to an IIS in case that the immune reaction to this pathogen was present or sufficient, thereby leading to a positive PSIIR. This reduced the definitely PSIIR-negative cohort to 300 patients. The demographic characteristics and CSF findings in PSIIR-positive compared to negative patients showed younger age, less impaired blood/CSF barrier, lower lactate levels as well as substantially increased quantitative intrathecal IgG synthesis (Supplementary Table 2). The frequencies of different diagnostic categories according to the case files as well as corresponding PSIIR status revealed that MS-S diseases represented the disease group most often diagnosed at discharge (32%) with the highest proportion of PSIIR positivity (41%, Supplementary Figure 1; Supplementary Table 3). Using the clinical and radiological data available at the time of the lumbar puncture (LP), in 51 of the PSIIR-positive 76 patients (67%), an MS-S disease could be confirmed (23–25) (Supplementary Table 4), while in the remaining 25 the re-analysis revealed that using the data available at LP the formal diagnosis of an MS-S disease (33%) could not be made (Non-MS-S, Figure 1).

**Figure 2:**
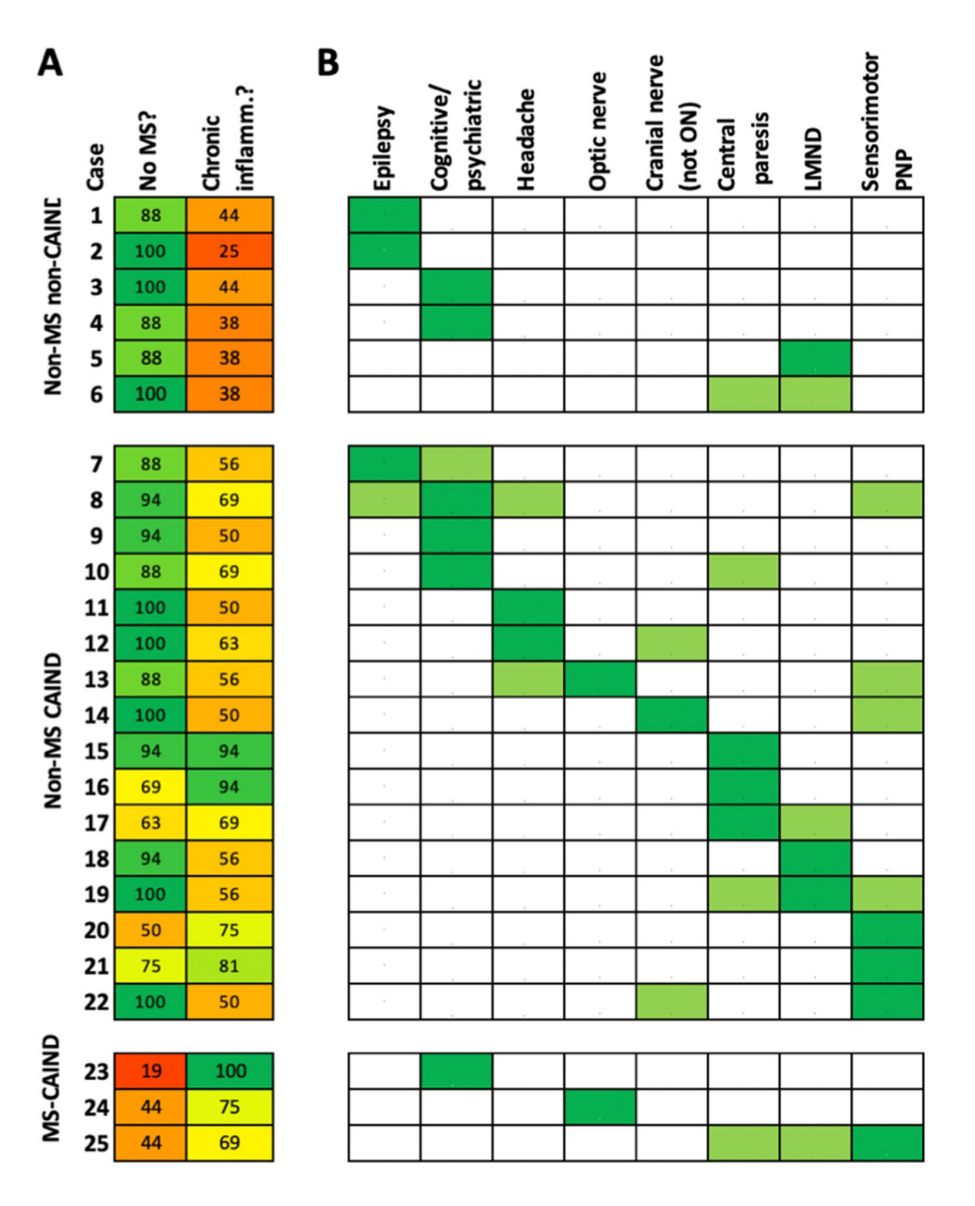
Clinical presentation of patients with non-MS spectrum diseases. Each of the 25 patients with positive PSIIR without diagnosis of an MS spectrum disorder is represented by one line. **(A)** Results of the Neuroimmunology expert rating. The numbers give the mean of the rating of the four experts (0 = definitely not, 100 = definitely yes) regarding the two survey statements: the patient does not have MS (no MS?), the patients suffers from a chronic autoimmune-inflammatory neurological disease (chronic inflamm?). The patients are grouped according to the survey results, not likely MS and not likely a chronic inflammatory disease (non-MS non-CAIND), not likely MS but likely a CAIND (non-MS CAIND), likely an CAIND but likely MS (MS-CAIND). The mean agreement score is color-coded (red: totally disagree, yellow: undecided, green: totally agree). **(B)** Symptoms repeatedly present in non-MS CAIND patients. When predominant these are indicated as dark green, when accompanying light green. Patients were grouped according to signs and symptoms for indicating involvement of different parts of the nervous system, cortical to peripheral anatomical localization from left to right. Cognitive/psychiatric: Cognitive decline and/or psychiatric symptoms, PNP: polyneuropathy, Cranial nerve (not ON): Other cranial neuropathy, not optic neuritis.

A neuroimmunology expert survey was employed to substantiate the assumption that the non-MS-S patients (a) indeed did not suffer from MS (no MS score, 100 = definitely not MS, 0 = definitely MS) but (b) from a CAIND (CAIND score, 100 = definitely a CAIND, 0 = definitely not a CAIND) using detailed case descriptions summarizing all information including all follow-ups available (case descriptions in the Appendix, Supplementary Table 5). In general, PSIIR-positive non-MS patients were rated less likely to suffer from MS (no MS score: mean 88, IQR 72–100) than from a CAIND (CAIND scores: median 56, IQR 47–72, *p* < 0.001, Supplementary Figure 2A). The higher the CAIND score, the more likely an MS diagnosis was rated (Supplementary Figure 2B). Consequently, six patients were rated rather unlikely suffer from a CAIND including MS (No-MS no-CAIND), three to have more likely MS than another CAIND (MS-CAIND). However, 16 patients remained in the group rated likely to indeed suffer from a CAIND other than MS (No-MS CAIND, Figure 2A).

### Non-MS CAIND PSIIR-positive patients exhibit diverse clinical phenotypes with less lesions suggestive for demyelination

The clinical presentations of the non-MS CAIND group were heterogenous and rarely convincingly classifiable using well-established diagnostic criteria for other neurological diseases (see eAppendix 1/2). The predominant neurological abnormalities comprised epilepsy (patient 7), encephalopathy with fluctuating consciousness and neuropsychiatric abnormalities (patient 8), multiple complaints interpreted as functional probably also due to neuropsychiatric abnormalities (patient 9), cognitive impairment associated with central tetraparesis (patient 10), headache (patients 11/12), optic neuropathy with dorsal column disorder (patient 13), vestibular neuropathy (patient 14), central monoparesis associated with CREST syndrome (patient 15), progressive spastic tetraparesis with sensory disturbances (patient 16/17), motor neuropathy associated with Sjögren’s syndrome (patient 18), ALS-like phenotype associated with psoriasis arthritis (patient 19), sensory mononeuritis multiplex associated with Crohn’s disease (patient 20), sensorimotor neuropathy with serology suggestive for Sjögren’s syndrome (patient 21) as well as facial hemispasm (patient 22). For a more systematic analysis eight frequent clinical components were identified. Patients were grouped according to their anatomical substrate from cerebral cortex for epileptic seizures to peripheral nerves for sensorimotor neuropathy (Figure 2B). Regarding the predominant symptoms, cognitive decline and/or psychiatric symptoms, central paresis suggesting spinal cord involvement and peripheral neuropathy were the most common symptoms (3/16, 19%). Including accompanying symptoms, peripheral neuropathy (7/16, 44%), central paresis (5/16, 31%), cognitive/psychiatric symptoms and headache (4/16 each, 25%) were most prevalent. More than half of the patients had symptoms of more than two or more of the eight categories (9/16, 56%, Figure 2B). Of note, three patients (19%) had lower motor neuron (LMN) involvement phenotypically similar to LMN disease (LMD), in two cases combined with central paresis mimicking amyotrophic lateral sclerosis (ALS). In 13 of the 16 non-MS CAIND patients (81%), follow-up data were available. These confirmed a chronic course of the disease in all 13 patients (Appendix, Supplementary Table 6). Of those treated with immunotherapy (*N* = 5), the majority showed a beneficial response (4/5, Appendix, Supplementary Table 6). Of the six PSIIR-positive patients categorized to rather not suffer from a CAIND, two each had epilepsy, cognitive decline, in one patient most likely Alzheimer’s disease, in both associated with cerebral ischemia, and two most likely ALS.

**Figure 3:**
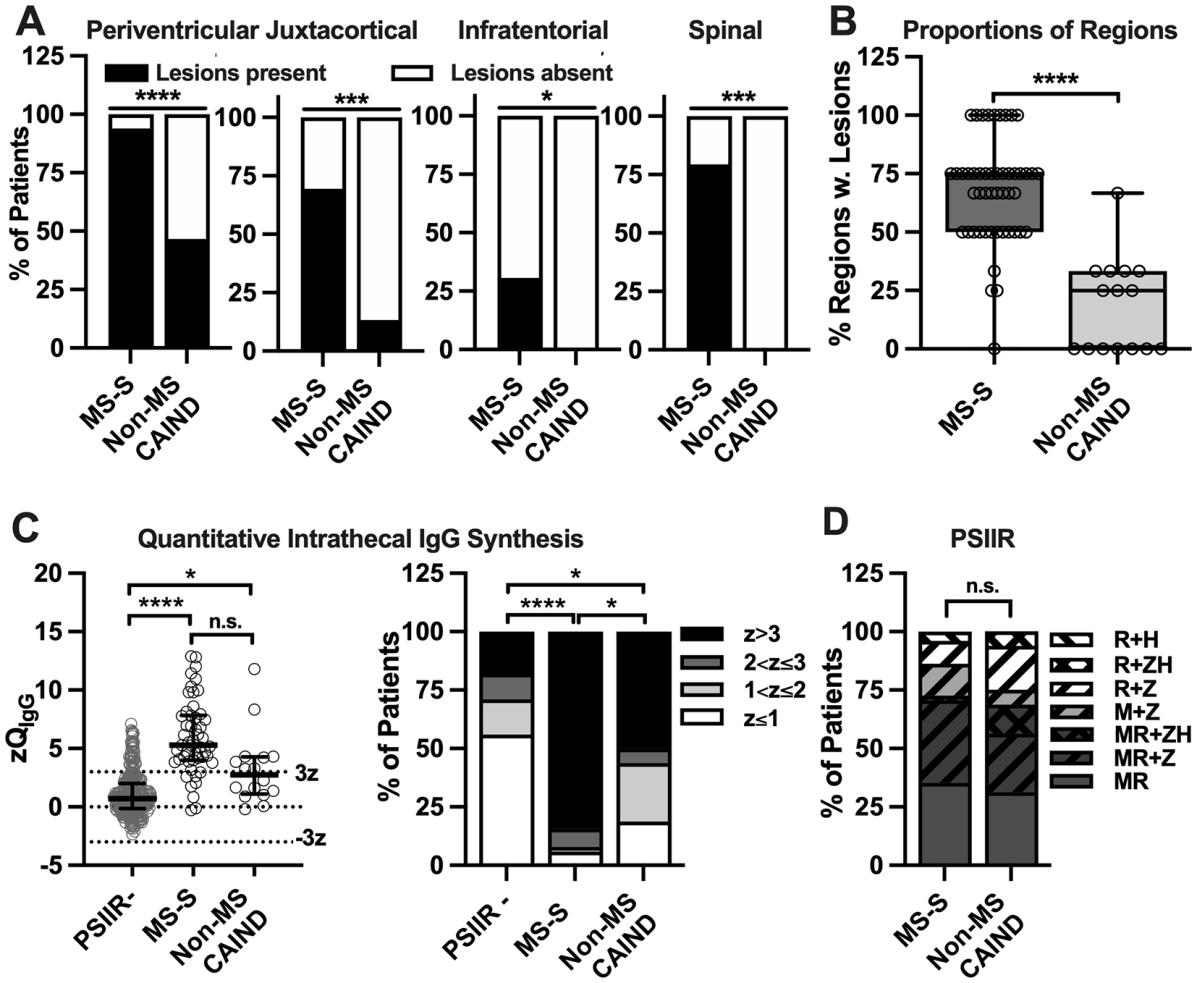
Less abundant demyelinating lesions and less frequent quantitative intrathecal IgG synthesis in non-MS chronic autoimmune-inflammatory neurological diseases with positive polyspecific intrathecal immune response (PSIIR compared to PSIIR-positive MS-S **(A)** Percentage of individual CNS regions with white matter lesions suggestive of demyelination among for patients with MR studies available in patients with non-MS chronic autoimmune-inflammatory neurological diseases (non-MS CAIND) compared to MS-spectrum diseases (MS-S). The regions are indicated above the graphs. **(B)** Percentage of regions with MR data available with lesions suggesting demyelination in non-MS CAIND and MS-S patients. The plots indicate the median, interquartile range and range of the percentages. **(C)** Left panel: Quantitative intrathecal IgG synthesis converted into Z scores. The left panel shows all PSIIR-negative patients (PSIIR-), PSIIR-positive MS-S as well as the non-MS CAIND patients. The normal range as mean plus/minus three standard deviation (z = 3/z = −3) is indicated by dotted lines. The right panel shows the same data categorized with z > 3 as definite intrathecal IgG synthesis (black), z > 2 and ≤ 3 as probable (dark grey), and z > 1 and ≤ 2 as possible (light grey) intrathecal IgG synthesis. **(D)** Proportion of increased pathogen-specific antibody indices (AI) contributing to the PSIIR. Background color indicates AIs from non-herpes viruses. Dark grey: measles + rubella AI increased (MR), light grey: only measles AI increased (M), white: only rubella AI increased. Oblique black stripes indicate increased herpes family virus AIs. From lower left to upper right: varicella zoster (Z) AI, from upper left to lower right: herpes simplex (H) AI. Statistical analysis was performed using **(A)** Fisher’s exact tests **(A)**, a Mann–Whitney *U* test **(B)**, Kruskal-Wallis test with Dunn’s multiple comparison’s post-tests (C, left panel), Chi-square test with *z* > 3 vs. z ≤ 3 (C, right panel), **(D)** Chi-square test MR vs. M or R, *p < 0.05, **p < 0.01, ***p < 0.001, ****p < 0.0001.

When comparing the group of non-MS CAIND with MS-S patients, no significant differences were observed regarding age and gender (Table 1). However, there was a tendency for a more balanced gender distribution in non-MS CAIND patients compared to the predominantly female MS-S group. Compared to MS-S patients, the proportion of demyelinating lesions in all four regions diagnostically relevant for MS was substantially lower in non-MS CAIND patients (Table 1; Figures 3A,B). Among symptoms typical for MS, we observed a tendency for optic neuritis to be less frequent in non-MS CAIND than in MS-S patients while headache tended to be more frequent in non-MS CAIND. Most notably, LMND occurred exclusively and peripheral sensorimotor neuropathy much more frequently in the non-MS CAIND group. Finally, when combined, systemic (Sjögren’s and CREST syndrome, psoriasis arthropathy) as well as organ-specific other autoimmune diseases, i.e., Crohn’s disease, atrophic gastritis and giant cell arteritis, occurred much more frequently in the non-MS CAIND group when compared to MS-S patients (Table 1).

**Table 1:** Clinical characteristics of PSIIR-positive non-MS patients with chronic inflammatory neurological disease compared to PSIIR-positive MS spectrum patients.

	MS-S (*N* = 51)	Non-MS CAIND (*N* = 16)	Statistics *p*-values
Demography			
Age (years), median (IQR)	58 (54–64)	63 (55–72)	0.199*
Female:male, *N* (%)	35:16 (69:31)	7:9 (44:56)	0.085
MS-typical symptoms and findings			
Optic neuritis, *N* (%)	12 (24)	1 (6)	0.165
Spastic parasparesis, *N* (%)	10 (20)	5 (31)	0.327
Regions w. demyel. (%), median (IQR)	75 (50–75)	25 (0–33)	<0.0001*
Symptoms not typical for MS			
Central nervous system			
Cognitive impairment, *N* (%)	15 (29)	3 (19)	0.527
Psychiatric symptoms, *N* (%)	5 (10)	4 (25)	0.201
Epilepsy, *N* (%)	4 (8)	2 (13)	0.623
Headache, *N* (%)	4 (8)	4 (25)	0.085
Peripheral nervous system			
Lower motor neuron disease, *N* (%)	0 (0)	3 (19)	0.012
Sensorimotor PNP, *N* (%)	3 (6)	7 (44)	0.001
Other associated diseases			
Neoplasm, *N* (%)	3 (6)	1 (6)	1.000
Autoimmune disease, *N* (%)	4 (8)	7 (44)	0.002

MS-S, MS spectrum; Non-MS CAIND, non-MS chronic inflammatory disease; Regions w. demyel, regions with demyelination; PNP, polyneuropathy; IQR, interquartile range; N, number. Statistical analysis was performed using Fisher’s exact test or Mann–Whitney U test (^*^). Two-tailed *p*-values are given. Significant differences are indicated with bold letters.

### CSF findings differ between PSIIR-positive non-MS and MS spectrum patients

Next, we evaluated whether CSF findings differed between PSIIR-positive MS-S and non-MS CAIND patients when compared with each other and the PSIIR-negative group as control (Table 2; Figures 3C,D). Although CSF cell count was slightly higher in the MS-S group compared to the two other groups, this difference was not significant. Blood-CSF barrier function measured as Q_Alb_Age_ was significantly less perturbed in MS-S when compared to PSIIR-negative patients, but not in non-MS CAIND. CSF lactate concentrations in MS-S group, but not in the non-MS CAIND group, were lower than in PSIIR-negative patients (Table 2). Most MS-S patients exhibited quantitative IIS for IgG, while IgG IIS was only present in one third of the non-MS CAIND patients (Figure 3C; Supplementary Table 7). When compared to PSIIR-negative patients, median zQ_IgG_ was 6.3-fold higher in the MS-S group but only 3.4-fold higher in the non-MS CAIND group. However, while definite quantitative IIS for IgG was significantly less frequent in non-MS CAIND compared to MS-S patients (Figure 3C, right panel, Supplementary Table 6), this difference did not quite reach statistical significance (*p* = 0.090, Supplementary Table 7). IIS for IgA and IgM were rare in all three groups (Supplementary Table 7). In summary, the main difference in the CSF findings in non-MS CAIND patients compared to the MS-S group was a substantially less pronounced quantitative IIS for IgG.

**Table 2:** Cerebrospinal fluid findings PSIIR-positive non-MS spectrum patients with chronic neurological inflammatory disease compared to PSIIR-positive MS spectrum and PSIIR-negative patients.

	PSIIR- (*N* = 300)	MS spectrum (*N* = 51)	Non-MS CAIND (*N* = 16)	Overall	MS-S vs. PSIIR-	Non-MS CAIND vs. PSIIR-	Non-MS CAIND vs. MS-S
Basic CSF variables				*p*-values			
Leukocytes/μL, median (IQR)	1 (1–3)	2 (1–6)	1 (0–3)	0.101	0.358	0.550	0.132
Leukocytes (>4/μL), *N* (%)	62 (21)	16 (31)	3 (19)	0.222^*^	0.305	1.000	1.000
Q_Alb_Age,_ median (IQR)	0.8 (0.6–1.1)	0.7 (0.6–0.9)	0.9 (0.6–1.0)	0.019	0.015	1.000	0.607
Q_Alb_Age_ ↑ (>1), *N* (%)	96 (32)	10 (20)	6 (38)	0.170^*^	0.294	1.000	0.547
Lactate (mmol/L), median (IQR)	1.8 (1.6–2.1)	1.6 (1.5–1.9)	1.7 (1.4–1.9)	<0.001	0.001	0.174	1.000
Lactate ↑ (>2.6 mmoL/L), *N* (%)	21 (7)	1 (2)	1 (13)	0.249^*^	0.669	0.983	0.418

PSIIR-, polyspecific intrathecal immune response negative; MS-S, MS spectrum; Non-MS CAIND, non-MS chronic inflammatory disease; CSF, cerebrospinal fluid; IQR, interquartile range; Q_Alb_, CSF/serum albumin ratio, Q_Alb_/Q_lim_, Q_Alb_ divided by age-dependent upper limit, Q_IgG/A//M_, CSF/serum IgG/A/M ratio. Z = Z score. IS, intrathecal synthesis. Statistical analysis for differences across all three groups were performed using Kruskal-Wallis or Chi-squared tests (^*^). Pair-wise statistical comparison was performed by Dunn’s post-test following Kruskal-Wallis test or Fisher’s exact test with Bonferroni’s correction following Chi-squared test. Two- tailed *p*-values are given. Significant differences are indicated with bold letters.

### The characteristics of the PSIIR in non-MS-S patients differ only slightly from those in MS-S patients

Finally, we analyzed whether the components of the PSIIR differ in PSIIR-positive MS-S and non-MS CAIND patients using the AIs of the PSIIR-negative group as a control. Measles, rubella and VZV had been measured in all patients. HSV AIs had been measured in only 35 and 38% of MS-S and non-MS-CAIND patients, respectively, less frequently than in PSIIR-negative patients (56%). This probably indicates that HSV-AI was ordered in addition to measles, rubella and VZV to exclude or confirm a PSIIR in patients finally remaining PSIIR-negative (Supplementary Table 8). The frequency of seronegativity or too low CSF titers to calculate individual AIs for some of the viruses tested was not different among all groups (Supplementary Table 8). When calculated according to Lange and Reiber (14), the frequency of elevated AIs among seropositive patients was significantly higher in both the MS-S and non-MS CAIND group compared to the PSIIR-negative control group for measles, rubella and VZV (Supplementary Table 9). No significant differences between non-MS CAIND and MS-S patients were detected. In contrast, when compared to the PSIIR-negative control group (11%), elevated HSV-AIs were detected more often in patients with non-MS CAIND (50%), while elevated HSV-AI occurred not more frequently in the MS-S group (18%, Supplementary Table 9). Overall, combinations of elevated AIs in MS-S and non-MS-CAIND patients did not differ regarding their frequency, except the combination HSV with VZV (Figure 3D; Supplementary Figure 3). The PSIIR in non-MS-CAIND more frequently comprised elevated AI for both VZV and HSV combined with either elevated measles or rubella AI or both (non-MS CAIND vs. MS-S: 3/16 (10%) vs. 1/51 (2%), *p* = 0.0396. Figure 3D). PSIIR-positivity due to an elevated HSV AI alone in combination in another non-herpes family virus AI occurred in one MS-S patient only and never in the non-MS CAIND group.

**Figure 4:**
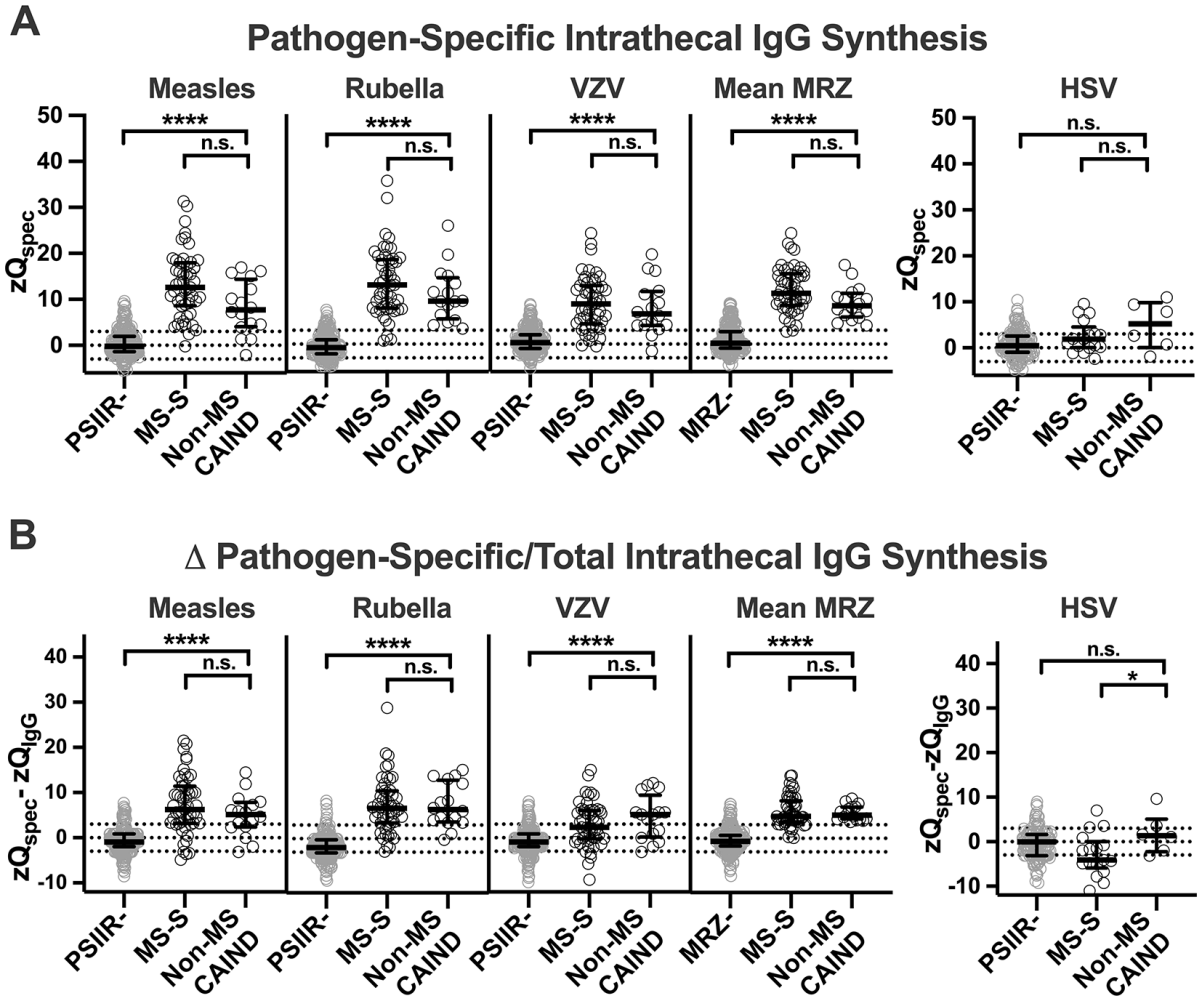
Patterns of the polyspecific intrathecal immune response (PSIIR) of non-MS patients with chronic inflammatory neurological diseases compared to patients with diseases of the MS spectrum and PSIIR-negative patients **(A)** Pathogen-specific intrathecal IgG synthesis against measles, rubella, Varicella zoster (VZV), and Herpes simplex (HSV) virus shown as Z score of the pathogen-specific Q_IgG_ (zQ_spec_). (A, left panel) The three left graphs show the individual zQ_spec_s for measles, rubella and Varicella zoster (VZV) for PSIIR-negative (PSIIR-), PSIIR-positive patients with MS-spectrum diseases (MS-S) or non-MS chronic autoimmune-inflammatory neurological diseases (non-MS CAIND). The right graph (MRZ) shows the mean Q_spec_ for each patient for all three viruses combined. Right panel: zQ_spec_ for HSV. **(B)** Predominance of pathogen-specific over total intrathecal IgG synthesis shown as difference of zQ_spec_ and zQ_IgG_ (Δ pathogen-specific/total intrathecal IgG synthesis, zQ_spec_ - zQ_IgG_). (A/B) The dotted lines indicate either Z = 3/−3 (3 SD below and above mean) and z = 0. Statistical analysis was performed using a Kruskal-Wallis test with Dunn’s multiple comparison’s post-tests. n.s., not significant, *p < 0.05, **p < 0.01, ***p < 0.001, ****p < 0.0001.

For CSF with quantitative IIS for total IgG (Q_IgG_ higher than the upper limit of normal, Q_lim_), the method for the pathogen-specific AI calculation according to Lange & Reiber switches its denominator from Q_IgG_ to Q_lim_ (14). Thus, pathogen-specific AI levels in groups with different frequencies of quantitative IIS for total IgG cannot be directly compared. Thus, to understand the differences in the PSIIR patterns observed in non-MS CAIND compared to MS-S patients, these needed to be calculated identically. The new IgG z score formula applied above to compare total IgG IIS of non-MS and MS-S CAINDs, for each Q_Alb_ creates an expected value (Q_mean_, z = 0) for the Q_IgG_ in the absence of IIS. Using this Q_mean_ as a denominator for the pathogen-specific CSF/serum ratio (Q_spec_) as well as the normalized standard deviation for total IgG, we calculated z scores for Q_spec_ (zQ_spec_, Figure 4A; Supplementary Table 10). As a proof of concept, the median zQ_spec_ for all for viruses was close to 0 in the PSIIR-negative cohort. The zQ_spec_s for measles, rubella and VZV were all significantly higher in the non-MS CAIND group compared to PSIIR-negative patients. However, although there was a tendency of higher zQ_spec_s in MS-S compared to non-MS CAIND patients, this difference did not reach significance for any of the three pathogens (Figure 4A, left panel). In contrast, the pattern of HSV was clearly different. The highest values of HSV-specific IgG IIS occurred in the non-MS CAIND group. However, this increase did not reach statistical significance, most probably due to the limited number of data points (Figure 4, right panel).

To get an idea, to what extend the pathogen-specific IgG IIS exceeds total IgG IIS, we substracted the corresponding zQ_IgG_ from each zQ_spec_ (zQ_spec_-zQ_IgG_). For measles, rubella and VZV, this resulted in quite similar and positive values for MS-S and non-MS CAIND patients. This finding indicated comparable predominance of pathogen-specific over total IgG IIS (Figure 4B, left panel, Supplementary Table 10). However, the median zQ_spec_-zQ_IgG_ for HSV became negative for MS-S patients and significantly lower than in the positive median zQ_spec_-zQ_IgG_ for HSV in non-MS CAIND group (Figure 4, right panel). Thus, in stark contrast to non-MS CAIND patients total IgG IIS exceeds HSV-specific IgG IIS in MS-S patients.

## Discussion

The main objective of this monocentric retrospective cross-sectional analysis was to identify PSIIR-positive CAINDs other than MS in an age group in which the onset of MS is rare. With one third of all PSIIR-positive patients, patients not formally meeting the diagnostic criteria of an MS-S disease were surprisingly frequent in our dataset. These patients often defied unequivocal diagnostic classification. Beyond the PSIIR, no broad CAIND biomarkers exist. Thus, we decided that rating by neuroimmunology experts whether those patients might suffer from a CAIND that most likely is not MS was the most adequate strategy to classify these patients in more detail. Indeed, for most PSIIR-positive patients not meeting MS-S diagnostic criteria the underlying disease was judged most likely to be a non-MS CAIND. The clinical presentation of these patients was quite heterogenous. It ranged from cognitive decline associated with headache suggestive for chronic encephalitis to chronically progressive spastic tetraparesis or peripheral sensorimotor neuropathy. Most notably, the clinical phenotype of some patients to some extent mimicked ALS. Compared to MS-S patients, MRI correlates of focal demyelination were considerably less frequent in non-MS CAIND patients. Therefore, in a minority of patients who clinically resembled PPMS, this diagnosis could not be made. Non-neurological autoimmune diseases were much more common in non-MS CAIND than in the MS-S group. As autoimmune diseases tend to co-occur (33, 34), this association further suggests the autoimmune etiology in this group.

Regarding CSF findings, a less frequent and less prominent quantitative IgG IIS was the main characteristic of non-MS-S CAIND compared to MS-S patients. Thus, the neurological phenotype, the associated non-neurological diseases, and the MRI and CSF characteristics in our cohort of non-MS PSIIR-positive non-MS CAIND patients confirmed that these patients are clearly distinct from MS-S patients. Albeit the quantitative IIS of total IgG was much less pronounced in the non-MS CAIND group when compared to MS-S patients, the pathogen-specific IIS of the PSIIR was not. Moreover, the combinations of AI elevations for the core PSIIR, measles, rubella and VZV, were quite similar to the MS-S group. The only exception was simultaneous pathogen-specific IIS of IgG directed against two herpes family viruses, VZV and HSV, which much more frequently in the non-MS CAIND group.

Taken together, we identified a group of clinically quite heterogenous, mostly unclassifiable PSIIR-positive CAINDs clearly distinct from MS-S. This finding was unexpected. Different defined CTDs like SLE (19, 20), Sjögren’s syndrome and granulomatosis with polyangiitis (20), when associated with nervous system involvement, have been reported to be PSIIR-positive in up to 20%. We recently described that autoimmune encephalitis with NMDA receptor antibodies exhibits a positive PSIIR in more than one third of the patients (21). Thus, we expected that those diseases will predominate in the group of non-MS-S PSIIR-positive patients. However, only one patient diagnosed Sjögren’s syndrome was identified. In another patient, the typically laboratory markers for Sjögren’s syndrome were found. Two others suffered from other systemic autoimmune disease, psoriatic arthritis and CREST syndrome, maybe indicating a broader range of systemic autoimmune diseases that can be associated with a PSIIR. Thus, diseases of the broader group of CTDs and CTD-related intractable disease (CTD-ID) (35) may have the propensity to develop a PSIIR upon nervous system involvement. Regarding CTDs, it must be kept in mind that many patients with CTD-typical clinical features do not meet the diagnostic criteria for one of the well-defined CTDs. These disease states are combined under the umbrella term undifferentiated CTD (UCTD). UCTDs account for up to 50% of all newly diagnosed CTDs (36). Indeed, neurological symptoms including convulsions, headache, and peripheral neuropathy, all present in our group of non-MS CAINDs, have been described in UCTD (37). However, none of the 13 non-MS CAIND patients without defined systemic autoimmunity had documented symptoms suggesting UCTD (38). In addition, as UCTD mainly affects young women (39), the demographics of our cohort were clearly different. However, the high frequency of UCTD among CTDs illustrates the fact that frequently autoimmune diseases cannot be classified. Based on our data, we conclude that those non-MS CAIND patients with no clinical or laboratory signs typical for a CTD may represent chronic organ-specific autoimmunities of the nervous system that like UCTD among CTDs defy diagnostic classification. At least in patient populations where MS is uncommon, these may occur quite frequently. We also propose that the PSIIR might serve as a suitable marker to screen for these disease states for further classification.

For MS, the most common CAIND subtype, the specificity of PSIIR positivity was reported to be about 90% (11, 18). In contrast, in >20% of our PSIIR-positive non-MS patients a CAIND was rated unlikely by our neuroimmunology experts. These patients more likely to have primarily neurodegenerative diseases or structural epilepsy. This contrasts the extremely low frequency of the PSIIR in non-autoimmune diseases reported previously (11). A possible explanation might be that an PSIIR without disease association becomes more frequent with age and thus the frequency observed in our study is due to the relatively high average age of our study population. Alternatively, neuroinflammation associated with neurodegeneration (40) or present in epilepsy (41) might have the propensity to induce a PSIIR.

The PSIIR is thought to result from a non-pathogen specific recruitment of memory B cells to specific niches in the chronically inflamed CNS where they antigen-independently proliferate and start antibody production (16, 17). Thus, frequently PSIIR-positive diseases should share a certain B-cell attracting and stimulating milieu. From this perspective, it seems puzzling why IIS specific to some viruses quite frequently contributes to a PSIIR while IIS specific to others with comparable seroprevalence does not (15). However, the typical temporal orchestration of the age of pathogen exposure with the age of onset of the autoimmune-mediated CNS inflammation in different diseases might be a defining factor. This might explain why the HSV AI clearly differed in our non-MS CAIND cohort when compared to MS-S patients.

The major limitation of our study is its retrospective nature regarding the analysis of CSF data. This might introduce a considerable bias as in only about half of patients with positive OCB aged 50 years or older the tests for the core AIs of the PSIIR were performed, probably guided by clinical suspicion of either MS or another suspected CAIND. In only half of these, the core AIs for measles, rubella and VZV were complemented by the HSV AI, which according to our analysis might be a marker to distinguish non-MS from MS-S CAIND. The fact that in PSIIR-negative patients, the additional HSV AI was ordered more often, clearly indicates that in part it was also measured to achieve PSIIR positivity. However, in the non-MS CAIND group, none of the PSIIRs relied on an elevated HSV AI. Thus, it can be assumed that PSIIR positivity is enriched in our patient sample as PSIIR measurement was performed more likely in CAIND patients who in turn will test positive more frequently. The other drawback is that only limited information of some of the patients, especially regarding follow-up, was available. Finally, to apply rating scores by neuroimmunology experts is a deliberately subjective tool that capitalizes on the clinical experience of the individual experts. This explains the considerable difference among the raters. In turn, misclassifications cannot be excluded. On the other hand, we believe that even considering these limitations, our dataset is still a valuable and suitable basis for the intended analysis, the identification and characterization of non-MS CAINDs in the elderly age group studied.

In conclusion, in elderly patients with positive OCBs and PSIIR a substantial proportion of diagnoses cannot be classified as a MS-S diseases. The majority of the PSIIR-positive non-MS-S patients most likely suffers from CAINDs clearly different from MS. Clinical presentations range from cortical symptoms like seizures and cognitive impairment to spinal cord, cranial and peripheral nerve and motor neuron involvement, often in combination. Our results strongly support our hypothesis that the PSIIR is a highly valuable biomarker to search for and identify new, currently still poorly defined non-MS CAINDs. Further studies with larger numbers of patients, comprehensive PSIIR testing and longitudinal investigations are needed to confirm our findings and to further detail the clinical presentation and course of non-MS CAINDs phenotype described in the study.

### Author information

HT, MSe, KHS, and FL were chosen as Neuroimmunology experts to rate individual case descriptions. All are board-certified Neurologists with focus on neuroimmunological diseases. HT is the head and MSe the deputy head of the neuroimmunology and multiple sclerosis outpatient and day clinic at the University Hospital Ulm. KHS and FL are both heads of the of the neuroimmunology and multiple sclerosis outpatient and day clinic of the University Hospital Schleswig-Holstein, Campus Kiel.

## Data availability statement

The original contributions presented in the study are included in the article/Supplementary material, further inquiries can be directed to the corresponding author.

## Ethics statement

The studies involving human participants were reviewed and approved by Ethikkommission der Universität Ulm. Written informed consent for participation was not required for this study in accordance with the national legislation and the institutional requirements.

## Author contributions

FB collected and analyzed data and wrote the first draft of the manuscript. DR, AH, and JD critically revised the manuscript including intellectually important content. VK and MSu collected data and critically revised the manuscript and contributed intellectually important content to the manuscript. MSe, FL, KHS, and HT performed the expert rating, critically revised the manuscript, and contributed intellectually important content to the manuscript. JL conceived the concept of the study, obtained the ethical review board approval, collected, and analyzed data, and finalized the manuscript. All authors contributed to the article and approved the submitted version.

## Funding

This study was supported by the German Federal Ministry of Education and Research (BMBF) through a grant Forschungsverbund CONNECT-GENERATE, grant code 01GM2208B.

## Conflict of interest

FB, DR, AH, JD, and VK report no conflict of interest. MSe has received consulting and/or speaker honoraria from Alexion, Bayer, Biogen, Bristol-Myers-Squibb, Merck, Roche, and Sanofi Genzyme. She has received travel support from Celgene, and TEVA. She has received research funding from the Hertha-Nathorff-Program. MSue reports personal fees and grants from Merck Healthcare Deutschland and Bayer Vital GmbH and grant support from the University of Greifswald (Gerhard-Domagk fellowship). KHS has received personal fees and travel grants from Bayer, Biogen, MerckSerono and Genzyme. FL received speaker fees, travel compensation and serves on advisory boards for/from Alexion, Bayer, Biogen, Fresenius, Merck-Serono, Novartis, Roche, Teva. His research is funded by German Federal Ministry of Education and Research (BMBF) and Deutsche Forschungsgesellschaft (DFG) and European Union (EU), outside the submitted work. HT reports funding for research projects, lectures and travel from Alexion, Bayer, Biogen, Celgene, Sanofi-Genzyme, Fresenius, Merck, Mylan, Novartis, Roche, Siemens Health Diagnostics, and Teva, and received research support from DMSG, DMS Stiftung, AMSEL-Stiftung Ursula Späth, Bayerische DMSG-Stiftung, Ministry of Science and Art of the State Baden-Württemberg (MWK-BW), and German Federal Ministry of Education and Research (BMBF). JL received speaker fees or travel compensation from UCB, Bayer, Roche, Teva and the Cure Huntington’s Disease Initiative (CHDI). His institution has been reimbursed for his role as a principal investigator in trials for UCB and CHDI. His research is funded by the European Huntington’s Disease Initiative and Ministry for Education and Research Baden-Württemberg outside the submitted work, as well as the German Federal Ministry of Education and Research (BMBF).

## Publisher’s note

All claims expressed in this article are solely those of the authors and do not necessarily represent those of their affiliated organizations, or those of the publisher, the editors and the reviewers. Any product that may be evaluated in this article, or claim that may be made by its manufacturer, is not guaranteed or endorsed by the publisher.

## Glossary

AI: antibody index
ALS: amyotrophic lateral sclerosis
AUC: area under the curve
CAIND: chronic autoimmune-inflammatory neurological disease
CCPD: combined central and peripheral demyelination
CIS: clinically isolated syndrome
CNS: central nervous system
CSF: cerebrospinal fluid
HSV: herpes simplex virus
IgA: immunoglobulin A
IgG: immunoglobulin G
IgM: immunoglobulin M
IIS: intrathecal immunoglobulin synthesis
LLQ: lower limit of quantification
LMND: lower motoneuron disease
LOMS: late-onset multiple sclerosis
MHC: major histocompactability complex
MRZ: measles, rubella, varicella zoster
MS: multiple sclerosis
MS-S: multiple sclerosis spectrum diagnosis
NMDAR-E: autoimmune encephalitis with NMDA receptor antibodies
OCB: oligoclonal bands
PPMS: primary progressive multiple sclerosis
PSIIR: polyspecific intrathecal immune response
Q_Alb_: CSF/serum albumin ratio
Q_Alb_Age_: age-adjusted Q_Alb_
Q_Alb_lim_: age-dependent upper Q_Alb_ limit of normal
Q_IgG_: CSF/serum IgG ratio
Q_lim_high_: upper normal limit of _QIgG_
RIS: radiologically isolated syndrome
ROC: receiver operator characteristics
RRMS: relapsing–remitting multiple sclerosis
SLE: systemic lupus erythematosus
ULN: upper limit of normal
VZV: varicella zoster
zQ_IgG_: Q_IgG_ Z score
