# Supplementary material for: Clinical associations and characteristics of the polyspecific intrathecal immune response in elderly patients with non-multiple sclerosis chronic autoimmune-inflammatory neurological diseases – a retrospective cross-sectional study
Source: Front Neurol. 2023 Jun 15;14:1193015. doi: 10.3389/fneur.2023.1193015 (PMC10311206; doi:10.3389/fneur.2023.1193015)
Supplement: Supplementary file 1 [file Data_Sheet_1.docx]

**Online Materials**

**Supplementary Methods**

**Generation of Z scores**

6,202 complete cerebrospinal fluid (CSF) results comprising CSF cell count, erythrocyte count, CSF/serum ratios of albumin (Q_Alb_), IgG, IgA and IgM (Q_IgG_, Q_IgA_, Q_IgM_), and oligoclonal bands) without obvious signs of inflammation (normal CSF cell count, no isolated oligoclonal bands, no obvious quantitative intrathecal immunoglobulin synthesis, Q_IgG_<Q_Alb_, Q_IgA_<Q_IgG_, Q_IgM_<Q_IgA_) and no relevant blood contamination (<1000 erythrocytes/µl) were identified from among CSF/serum pairs measured in two CSF lab during an episode of 10 years using BNProspec Nephelometers (Siemens Healthcare GmbH, Erlangen, Germany). Datasets with extreme serum albumin and IgG concentrations were excluded using the ROUT test (1%) from GraphPad Prism (Graphpad Software Inc., La Jolla, CA, US). In addition, all datasets with serum IgG lower than 0.5-fold the lower level of normal were removed (Supplementary Figure 1 A/B). For those of the remaining datasets with a Q_Alb_ between 2.0 x 10^-3^ and 15 x 10^-3^ (N=5,553), all Q_IgG_ transformed using the natural logarithm (LN) were plotted against the LN-transformed Q_Alb_ using the GraphPad Prism Software (Supplementary Figure 2A). Linear regression revealed a highly linear relationship between LN(Q_IgG_x10^3^) and LN(Q_Alb_x10^3^) (R squared 0.9236) following the formula 1.

*Formula 1: LN(Q_IgG_* *x10^3^)=0.9698*LN(Q_Alb_* *x10^3^)-0.686*3

All LN(Q_IgG_) values were corrected to the median LN(Q_Alb_), which was 1.77 (interquartile range 1.40-2.06) which coincided with the mean (1.77, standard deviation 0.41) using formula 2 (Supplementary Figure 2B, left panel).

*Formula 2: Adjusted LN(Q_IgG_ x10^3^)=(1.77- LN(Q_Alb_ x10^3^)*0.9698+ LN(Q_IgG_ x10^3^)*

After removal of 117 outliers using the ROUT algorithm set to 1%, the resulting distribution of the adjusted LN(Q_IgG_ x10^3^) showed remarkably symmetrical distribution with a skewness (0.3) with a kurtosis of 0.7 (Gaussian distribution 0) with a mean of 1.03 and a SD of 0.114 (Supplementary Figure 2C).

For conversion of QIgG into Z scores (zQIgG) we thus used the following formula:

Formula 3: Z Q_IgG_=(*LN(Q_IgG_ x10^3^)*-(0.9698**LN(Q_Alb_ x10^3^)*-0.6863))/0.114

When converting the original dataset, this resulted a symmetrical distribution of the Z score around the expected mean of 0 from a Q_Alb_ of 2.0x10^-3^ to 15.0x10^-3^ when using smoothing spline interpolation with 4 knots (Supplementary Figure 2D). To further prove that the Z score conversion results in meaningful results across the range of Q_Alb_s, we evaluated the distribution of Z scores of after categorization into six groups, 2**≤**Q_Alb_x10^3^<3, 3**≤**Q_Alb_x10^3^<5, 5**≤**Q_Alb_x10^3^<7, 7**≤**Q_Alb_x10^3^<9, 9**≤**Q_Alb_x10^3^<12, 12**≤**Q_Alb_x10^3^<15.

| **Q_Alb_x10^3^** | **2-3** | **3-5** | **5-7** | **7-9** | **9-12** | **12-15** |
| --- | --- | --- | --- | --- | --- | --- |
| N | 290 | 1673 | 1695 | 1037 | 628 | 229 |
| Mean | 0.082 | 0.025 | -0.053 | -0.023 | 0.024 | 0.200 |
| SD | 1.040 | 1.020 | 0.959 | 0.955 | 0.992 | 1.260 |

***Supplementary Methods Table1****: Q_IgG_ Z score means and standard deviation (SD) when categorized according to the Q_Alb_.*

This indicated that the mean as well as the SD remained remarkably stable in the range of Q_Alb_s used to generate the formula to generate Z scores. Thus, those can be used as an indicator of intrathecal synthesis of IgG, which can be categorized as proven when Z>3, and to compare the intrathecal synthesis of IgG across different CSF/serum pairs with different Q_Alb_s in the range used to generate the algorithm. We deliberately categorized IIS as possible when the zQ_IgG_ was >1 and **≤**2, probable when >2 and **≤**3.

zQ_spec_s were calculated accordingly. First, the Q_spec_ was regenerated from the antibody indices (AI) by multiplying the AI with Q_IgG_ when Q_IgG_**≤**Q_lim_, of multiplied by the Q_lim_ when Q_IgG_**>**Q_lim._ Then, Formula 3 was applied with Q_spec_ instead of Q_IgG_.


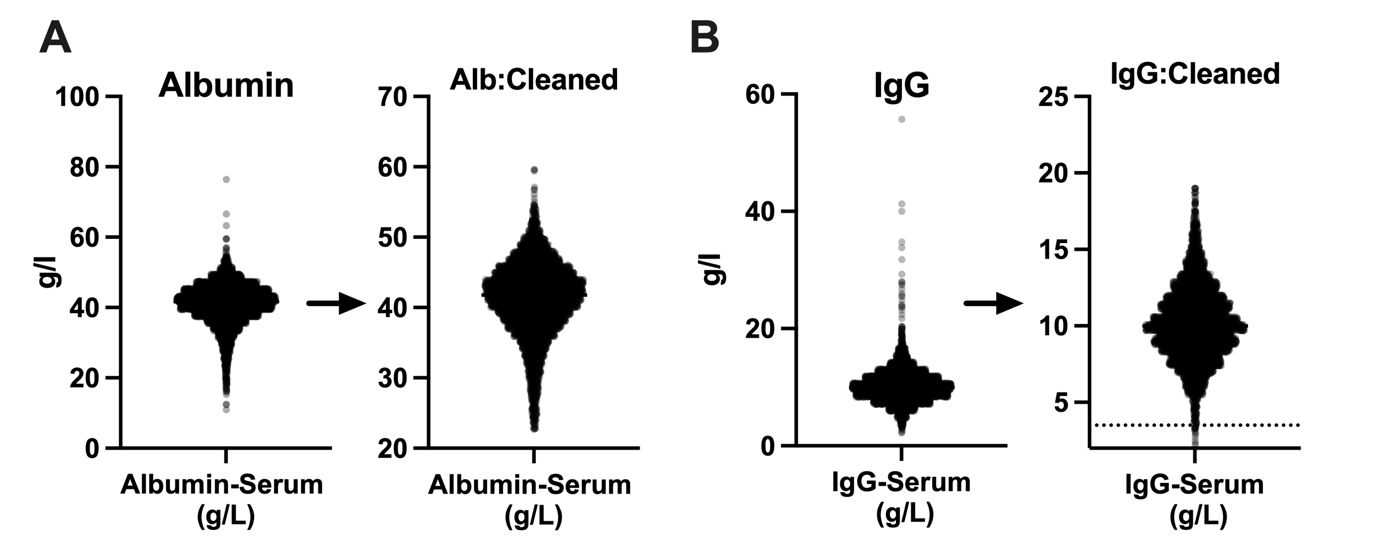


**Supplementary Methods Figure 1: Filtering of the dataset of 6202 CSF/serum pairs by serum albumin and IgG concentration.** Extreme outliers were removed from the dataset using the ROUT algorithm (Q=1%) which led to the removal of 57 datasets via albumin and 36 dataset via IgG. Additional datasets were excluded due to a serum IgG lower than 0.5-fold the lower level of normal (dotted line).


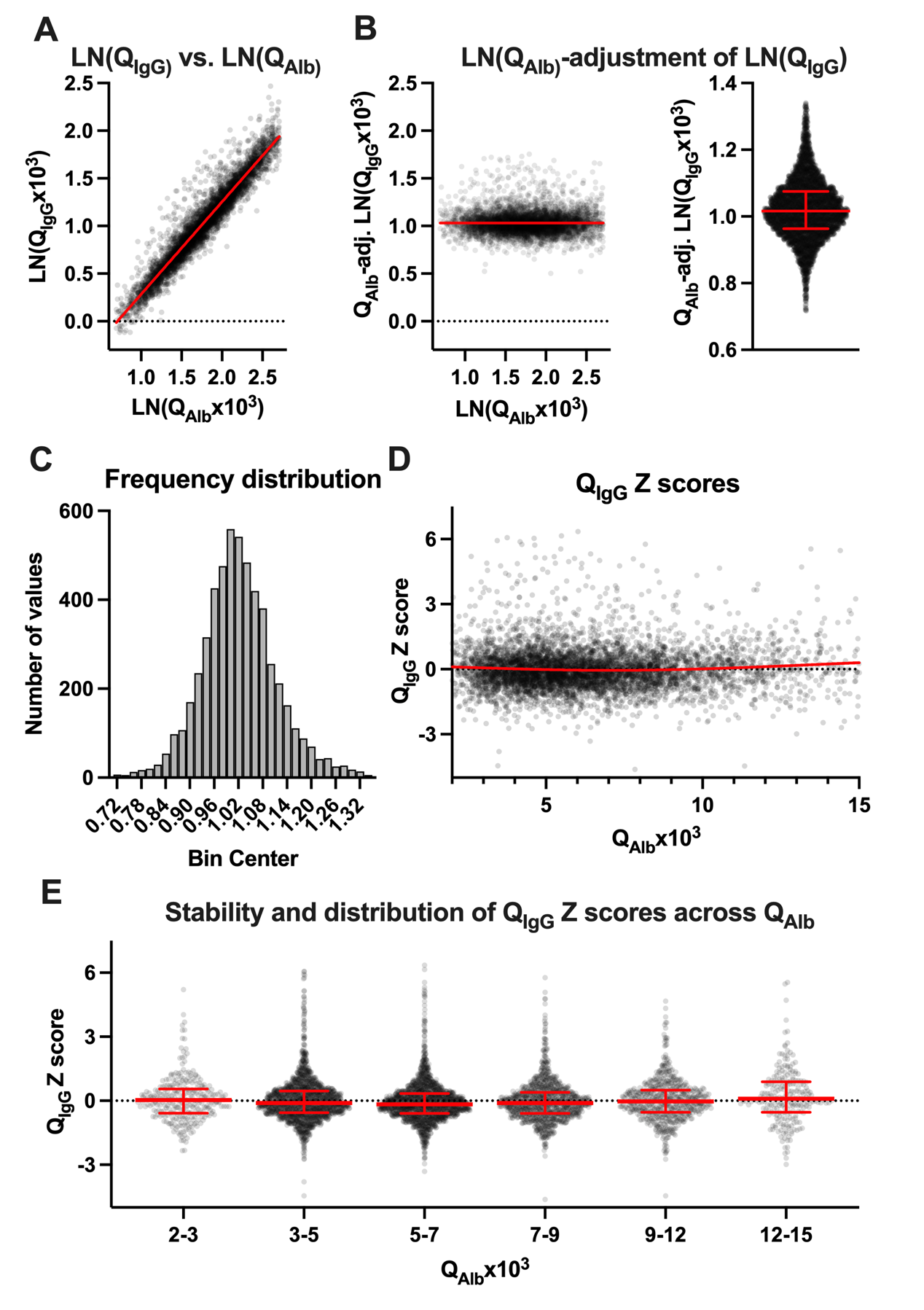


**Supplementary Methods Figure 2: Generation and testing of Z score conversion using 5553 paired CSF and serum albumin and IgG datasets without evidence of inflammation or blood contamination.** (A) logarithmized CSF/serum IgG and albumin ratios after multiplication with 1000 (LN(Q_IgG_x10^3^) plotted against the logarithmized CSF/serum albumin ratio after multiplication with 1000 (LN(Q_Alb_x10^3^)). (B) Adjustment of the LN(Q_IgG_x10^3^) to the mean LN(Q_Alb_x10^3^. (C) Distribution of Q_Alb_-adjusted LN(Q_IgG_x10^3^). (D/E) The distribution of Z scores across the range of Q_Alb_. Red lines indicate results of linear regression (A/B), median and SD (B, right panel/E) and smoothing spline interpolation (D).

**Expert Rating**

Four experts of neuroimmunology (F.L., K.S., M.Se., H.T.) were provided with the case description in the Appendix 1 and the tabular case summary of Appendix 2. In addition, each was provided with an Excel sheet with the following statements to be rated for each patient:

*Statement 1: The patients does not suffer from MS*

*Statement 2: The patient suffers from a chronic inflammatory disease of the nervous system*

The statements could be rated from “I totally agree (100)”, “I partially agree (75)”, “undecided (50)”, “I rather disagree (25)” to “I totally disagree (0)”. The raw results when converted to a scale from 0 to 100 as well as the means are shown in Supplementary Table 5. The means are shown color-coded in Figure 2.

**Supplementary Data**

**Supplementary Tables**

**Supplementary Table 1** | Availability of MRI data for re-analysis

|  | **MS-S**  **(N=51)** | **Non-MS-CAIND**  **(N=16)** | **Non-MS non-CAIND**  **(N=6)** | **MS? CAIND**  **(N=3)** | **MS-S vs.**  **non-MS-CAIND** | **MS-S vs Non-MS non-CAIND** | **MS-S vs. MS? CAIND** |
| --- | --- | --- | --- | --- | --- | --- | --- |
| **N (%)** | | | | | **P-values** | | |
| Cerebral MRI | 49(96) | 15(94) | 6 (100) | 3 (100) | 0.57 | 1.00 | 1.00 |
| Spinal MRI | 36(71) | 7(44) | 2 (33) | 2 (67) | 0.07 | 0.09 | 1.00 |

MS-S: Multiple sclerosis spectrum, CAIND: Chronic autoimmune-inflammatory nervous system disease

**Supplementary Table 2 I** Characteristics of PSIIR-negative compared with PSIIR-positive patients

|  | **PSIIR-negative**  **(N=300)** | **PSIIR-positive**  **(N=76)** | **Statistics** |
| --- | --- | --- | --- |
| **Demography** |  |  | **p values** |
| **Age (years),** median (ICR) | 64 (56-73) | 59 (54-68) | **<0.01** |
| **Female:male,** N (%) | 165:135 (55:45) | 46:30 (61:39) | 0.44 |
| **Basic CSF variables** |  |  |  |
| **Leukocytes/μl,** median (IQR) | 1 (1-3) | 2 (1-5) | 0.52 |
| **Leukocytes (>4/μl)**, N (%) | 62 (21) | 21 (28) | 0.22 |
| **Q_Alb_/Q_lim,_** median (IQR) | 0.8 (0.6-1.1) | 0.7 (0.6-0.9) | **<0.01** |
| **Q_Alb_/Q_lim_ ↑ (>1)**, N (%) | 96 (32) | 18 (24) | 0.17 |
| **Lactate (mmol/l),** median (IQR) | 1.8 (1.6-2.1) | 1.6 (1.5-1.9) | **<0.0001** |
| **Lactate ↑ (>2.6mmol/l),** N (%) | 21 (7) | 3 (4) | 0.44 |
| **Quant. intrathecal Ig synthesis** |  |  |  |
| **Z Q_IgG_,** median (IQR) | 0.8 (-0.1 to 2.3) | 4.4 (2.3-6.6) | **<0.0001** |
| **IgG IS,** N (%) | 41 (14) | 49 (64) | **<0.0001** |
| **IgA IS,** N (%) | 11 (4) | 2 (3) | 1.00 |
| **IgM IS,** N (%) | 12 (5) | 4 (7) | 0.54 |

PSIIR: polyspecific intrathecal immune response, CSF: cerebrospinal fluid, IQR: interquartile range, Q_Alb_: CSF/serum albumin ratio, Q_Alb_/Q_lim_: Q_Alb_ divided by age-dependent upper limit, Q_IgG/A//M_: CSF/serum IgG/A/M ratio. Z = Z score. IS: intrathecal synthesis. ^§^CSF IgM negative in 59 PSIIR-negative and 15 PSIIR-positive patients. Statistical analysis was performed by Whitney-Mann or Fisher’s exact test. Two-tailed p-values are given. Significant differences are indicated with bold letters and grey background.

**Supplementary Table 3** | **Positivity of the polyspecific intrathecal immune response**

| **Disease group (N=415)**  **(N, %)** | **PSIIR + (N=76)**  **N(%)** | **PSIIR? (N=39)**  **N(%)** | **PSIIR – (N=300)**  **N(%)** |
| --- | --- | --- | --- |
| MS-S (134, 32%) | 55(41%) | 8 (6%) | 71 (53%) |
| Other AIND (47, 11%) | 1 (2%) | 3 (6%) | 43 (91%) |
| Neurodegenerative (57, 14%) | 5 (9%) | 7 (12%) | 45 (79%) |
| Peripheral/cranial nerve (31, 7%) | 2 (6%) | 3 (10%) | 26 (84%) |
| Infectious (28, 7%) | 3 (11%) | 4 (14%) | 21 (75%) |
| Cerebrovasc. (26, 6%) | 2 (8%) | 4 (15%) | 20 (77%) |
| Epilepsy (23, 6%) | 3 (13%) | 2 (9%) | 18 (78%) |
| Neoplasm (10, 2%) | 0 (0%) | 1 (10%) | 9 (90%) |
| Headache/ facial pain (10, 2%) | 2 (20%) | 2 (20%) | 6 60%) |
| Other (49, 12%) | 3 (6%) | 5 (10%) | 41 (84%) |

MS-S = MS spectrum diseases: all MS subtypes, clinically isolated syndrome, radiologically isolated syndrome. Other AIND = Other autoimmune-inflammatory neurological diseases, Neurodegenerative = Neurodegenerative diseases, Peripheral nerve = isolated peripheral or cranial nerve lesions, Infectious = infectious neurological diseases, Cerebrovasc. = cerebrovascular diseases, Other = other neurological diseases, PSIIR = polyspecific intrathecal immune response, PSIIR+ = PSIIR positive, PSIIR? = PSIIR unclear (to many antibody indices uncalculatable or increased due to CSF/plasma disequilibrium or infectious), PSSIR- = PSIIR negative.

**Supplementary Table 4 |** Patients with MS spectrum diseases: disease subtypes, demographic characteristics, number of regions with demyelinating lesions upon MRI and number of patients with late-onset MS.

|  | **N**  **(%)** | **Age (years), median, ICR** | **Female:male**  **N (%)** | **LOMS**  **N (%)** |
| --- | --- | --- | --- | --- |
| RRMS | 21 (41) | 56 (53 - 63) | 15:6 (71:29) | 16 (76) |
| PPMS | 15 (29) | 62 (55 - 68) | 9:6 (60:40) | 13 (87) |
| SPMS | 8 (16) | 59 (52 - 74) | 5:3 (63:37) | 2 (25) |
| RIS | 4 (8) | 59 (53 - 60) | 4:0 (100:0) | - |
| CIS | 3 (6) | 55 (55 - 58) | 2:1 (67:33) | - |
| Total | 51 (100) | 58 (54 - 64) | 35:16 (69:31) | 31 (70) |

MS: multiple sclerosis, RRMS: relapsing-remitting MS, PPMS: primary progressive MS, SPMS: secondary progressive MS, RIS: radiologically isolated syndrome, CIS: clinically isolated syndrome, N: number of patients, IQR: interquartile range, LOMS: late-onset MS. The percentage of LOMS refers to MS patients only.

**Supplementary Table 5** | Results of the expert rating

|  | Case | No MS | | | | | | | Chronic inflammatory CNS Disease | | | | | | | | |
| --- | --- | --- | --- | --- | --- | --- | --- | --- | --- | --- | --- | --- | --- | --- | --- | --- | --- |
| Rater | | 1 | | 2 | | 3 | 4 | Mean | | 1 | | 2 | 3 | | 4 | | Mean |
| Non-MS  Non-CAIND | 1 | 50 | 100 | | 100 | | 100 | 88 | 50 | | 25 | | 0 | 100 | | 44 | |
|  | 2 | 100 | 100 | | 100 | | 100 | 100 | 25 | | 50 | | 0 | 25 | | 25 | |
|  | 3 | 100 | 100 | | 100 | | 100 | 100 | 0 | | 75 | | 0 | 100 | | 44 | |
|  | 4 | 75 | 100 | | 75 | | 100 | 88 | 25 | | 100 | | 0 | 25 | | 38 | |
|  | 5 | 50 | 100 | | 100 | | 100 | 88 | 25 | | 75 | | 25 | 25 | | 38 | |
|  | 6 | 100 | 100 | | 100 | | 100 | 100 | 25 | | 75 | | 25 | 25 | | 38 | |
| Non-MS  CAIND | 7 | 75 | 100 | | 75 | | 100 | 88 | 25 | | 75 | | 25 | 100 | | 56 | |
|  | 8 | 75 | 100 | | 100 | | 100 | 94 | 50 | | 100 | | 25 | 100 | | 69 | |
|  | 9 | 75 | 100 | | 100 | | 100 | 94 | 50 | | 75 | | 50 | 25 | | 50 | |
|  | 10 | 50 | 100 | | 100 | | 100 | 88 | 75 | | 100 | | 25 | 75 | | 69 | |
|  | 11 | 100 | 100 | | 100 | | 100 | 100 | 25 | | 50 | | 25 | 100 | | 50 | |
|  | 12 | 100 | 100 | | 100 | | 100 | 100 | 25 | | 50 | | 75 | 100 | | 63 | |
|  | 13 | 75 | 75 | | 100 | | 100 | 88 | 25 | | 100 | | 25 | 75 | | 56 | |
|  | 14 | 100 | 100 | | 100 | | 100 | 100 | 25 | | 100 | | 0 | 75 | | 50 | |
|  | 15 | 75 | 100 | | 100 | | 100 | 94 | 75 | | 100 | | 100 | 100 | | 94 | |
|  | 16 | 50 | 25 | | 100 | | 100 | 69 | 75 | | 100 | | 100 | 100 | | 94 | |
|  | 17 | 50 | 100 | | 0 | | 100 | 63 | 75 | | 50 | | 50 | 100 | | 69 | |
|  | 18 | 75 | 100 | | 100 | | 100 | 94 | 50 | | 100 | | 0 | 75 | | 56 | |
|  | 19 | 100 | 100 | | 100 | | 100 | 100 | 25 | | 75 | | 25 | 100 | | 56 | |
|  | 20 | 50 | 75 | | 25 | | 50 | 50 | 50 | | 100 | | 50 | 100 | | 75 | |
|  | 21 | 50 | 75 | | 75 | | 100 | 75 | 50 | | 100 | | 75 | 100 | | 81 | |
|  | 22 | 100 | 100 | | 100 | | 100 | 100 | 25 | | 100 | | 25 | 50 | | 50 | |
| MS?  CAIND | 23 | 25 | 0 | | 25 | | 25 | 19 | 100 | | 100 | | 100 | 100 | | 100 | |
|  | 24 | 25 | 0 | | 75 | | 75 | 44 | 75 | | 100 | | 25 | 100 | | 75 | |
|  | 25 | 50 | 0 | | 25 | | 100 | 44 | 50 | | 100 | | 25 | 100 | | 69 | |

**Supplementary Table 6** | Follow-up and response to immunotherapy

|  | **Non-MS**  **Non-CAIND** | **Non-MS**  **CAIND** | **MS CAIND** |
| --- | --- | --- | --- |
| **Neurological follow-up available**, N/N (%) | 0/6 (0) | 13/16 (81) | 0/3 (0) |
| **Years of follow-up**, median (IQR) | - | 5.8 (2.9-8.3) | - |
| **Chronic disease**, N/N (%) | - | 13/16 (81) | - |
| **MS upon follow-up reported,** N/N (%) | 0/2 (0) | 1/13 (8) | 1/3 (33) |
| **Immunosuppressive treatment,** N/N (%) |  | 5/16 (31) |  |
| **Response to immunosuppression**, N/N (%) |  | 4/5 (80) |  |

MS: multiple sclerosis, only mentioned as diagnosis in report from non-neurological treatments.

**Supplementary Table 7 | Cerebrospinal fluid findings PSIIR-positive non-MS spectrum patients with chronic inflammatory disease compared to PSIIR-positive MS spectrum and PSIIR-negative patients**

|  | **PSIIR-**  **(N=300)** | **MS spectrum**  **(N=51)** | **Non-MS CAIND**  **(N=16)** | **Overall** | **MS-S vs. PSIIR-** | **Non-MS CAIND vs. PSIIR-** | **Non-MS CAIND vs. MS-S** |
| --- | --- | --- | --- | --- | --- | --- | --- |
| **Quant. intrathecal Ig synthesis** | | | | p-values | | | |
| **Z Q_IgG_,** median (IQR) | **0.8 (-0.1 to 2.3)** | **5.0 (4.0 to 7.8)** | **2.7 (1.3 to 4.2)** | **<0.0001** | **<0.0001** | **0.026** | 0.090 |
| **IgG IS,** N (%) | **41 (14)** | **41 (80)** | **5 (31)** | **<0.0001*** | **<0.0001** | 0.198 | **0.002** |
| **IgA IS,** N (%) | 11(4) | 1 (2) | 1 (6) | 0.694* | 1.000 | 1.000 | 1.000 |
| **IgM IS,** N (%) | 12 (4) | 3 (6) | 1 (7) | 0.290* | 1.000 | 0.693 | 1.000 |

PSIIR-: polyspecific intrathecal immune response negative, MS-S: MS spectrum, Non-MS CAIND: non-MS chronic inflammatory disease, CSF: cerebrospinal fluid, IQR: interquartile rangeQ_IgG/A//M_: CSF/serum IgG/A/M ratio. Z = Z score. IS: intrathecal synthesis. Statistical analysis for differences across all three groups were performed using Kruskal-Wallis or Chi-squared tests (*). Pair-wise statistical comparison was performed by Dunn’s post-test following Kruskal-Wallis test or Fisher’s exact test with Bonferroni’s correction following Chi-squared test. Two- tailed p-values are given. Significant differences are indicated with bold letters.

**Supplementary Table 8 | Comparison of non-MS spectrum CAIND patients with MRZ-negative and MS spectrum patients:** Proportion of patients with antibody indices for measles, rubella, VZV and HSV measured, and those with positive serology enabling AI calculation according to Lange and Reiber

|  | **PSIIR-** | **MS-S** | **Non-MS CAIND** | **Overall** | **MS-S vs. PSIIR-** | **Non-MS CAIND vs. PSIIR-** | **Non-MS CAIND vs. MS-S** |
| --- | --- | --- | --- | --- | --- | --- | --- |
| **AI measured** | | | | **p-values** | | | |
| **Measles AI, N/N (%)** | 300/300 (100) | 51/51 (100) | 16/16 (100) | 1.0000* | 1.000 | 1.000 | 1.000 |
| **Rubella AI, N/N (%)** | 300/300 (100) | 51/51 (100) | 16/16 (100) | 1.0000* | 1.000 | 1.000 | 1.000 |
| **VZV AI, N/N (%)** | 300/300 (100) | 51/51 (100) | 16/16 (100) | 1.0000* | 1.000 | 1.000 | 1.000 |
| **HSV AI, N/N (%)** | **167/300 (56)** | 18/51 (35) | 6/16 (38) | **0.0131** | **0.028** | 0.597 | 1.000 |
| **AI measurable** | | | | **p-values** | | | |
| **Measles AI, N/N (%)** | 289/300 (96) | 51/51 (100) | 16/16 (100) | 0.2268* | 1.000 | 1.000 | 1.000 |
| **Rubella AI, N/N (%)** | 279/300 (93) | 50/51 (98) | 16/16 (100) | 0.1485* | 0.669 | 1.000 | 1.000 |
| **VZV AI, N/N (%)** | 299/300 (99) | 51/51 (100) | 16/16 (100) | 1.000* | 1.000 | 1.000 | 1.000 |
| **HSV AI, N/N (%)** | 157/167 (94) | 17/18 (94) | 6/6 (100) | 1.000* | 1.000 | 1.000 | 1.000 |

PSIIR-: polyspecific intrathecal immune response negative, MS-S: MS spectrum, Non-MS CAIND: non-MS chronic inflammatory disease, IQR: interquartile range, AI: CSF/serum antibody index, M: measles, R: rubella, Z: Varicella zoster, H: Herpes simplex. Q_spec_: pathogen-specific CSF/ serum IgG ratio, Q_IgG_: total CSF/ serum IgG ratio. N: number, IQR: interquartile range. Statistical analysis for differences across all three groups were performed using Chi-squared tests or in case that data did not meet the criteria for a Chi-squared test using a Fisher’s exact test comparing PSIIR-negative or -positive patients (*). Pair-wise statistical comparison was performed as Fisher’s exact test with Bonferroni’s correction following global testing. Two-tailed p-values are given. Significant differences are indicated with bold letters.

**Supplementary Table 9 | Comparison of non-MS spectrum CAIND patients with MRZ-negative and MS spectrum patients:** Proportion of elevated antibody indices for measles, rubella, VZV and HSV in patients with positive serology calculated according to Lange and Reiber

|  | **PSIIR-**  **(N=300)** | **MS-S**  **(N=51)** | **Non-MS CAIND**  **(N=16)** | **Overall** | **MS-S vs. PSIIR-** | **Non-MS CAIND vs. PSIIR-** | **Non-MS CAIND vs. MS-S** |
| --- | --- | --- | --- | --- | --- | --- | --- |
| **AI according to Reiber & Lange ↑(≥1.5), N/N (%)** | | | | **p-values** | | | |
| **M** | **29/289 (10)** | 44/51 (86) | 12/16 (75) | **<0.0001** | **<0.0001** | **<0.0001** | 0.438 |
| **R** | **14/279 (5)** | 44/50 (88) | 15/16 (94) | **<0,0001** | **<0.0001** | **<0.0001** | 1.000 |
| **Z** | **37/297 (12)** | 31/51 (61) | 11/16 (69) | **<0.0001** | **<0.0001** | **<0.0001** | 0.768 |
| **H** | 17/157 (11) | 3/17 (18) | **3/6 (50)** | **0.015** | 0.420 | **0.025** | 0.279 |

PSIIR-: polyspecific intrathecal immune response negative, MS-S: MS spectrum, Non-MS CAIND: non-MS chronic inflammatory disease, IQR: interquartile range, AI: CSF/serum antibody index, M: measles, R: rubella, Z: Varicella zoster, H: Herpes simplex. Q_spec_: pathogen-specific CSF/ serum IgG ratio, Q_IgG_: total CSF/ serum IgG ratio. N: number, IQR: interquartile range. Statistical analysis for differences across all three groups were performed Chi-squared tests. Pair-wise statistical comparison was performed as Dunn’s post-test following Kruskal-Wallis test or Fisher’s exact test with Bonferroni’s correction following Chi-squared test. Two-tailed p-values are given. Significant differences are indicated with bold letters.

**Supplementary Table 10 | Comparison of non-MS spectrum CAInD patients with MRZ-negative and MS spectrum patients:** Antibody indices for measles, rubella, VZV and HSV calculated as z-scores as well as the difference of virus-specific and total IgG z-scores indicating a predominantly virus-specific intrathecal IgG synthesis

|  | **PSIIR-**  **(N=300)** | **MS-S**  **(N=51)** | **Non-MS CAIND**  **(N=16)** | **Overall** | **Non-MS-CAIND vs. PSIIR-** | **Non-MS CAIND vs. MS-S** |
| --- | --- | --- | --- | --- | --- | --- |
| **Z Score Q_spec_, median (IQR), N** | | | | **p-values** | | |
| **M** | **0.2 (-1.2 to 2.1), 289** | 12.6 (8.7 to 17.6), 51 | 7.7 (4.4 to 12.9), 16 | **<0.0001** | **<0.0001** | 0.346 |
| **R** | **-0.6 (-2.1 to 1.2), 279** | 12.9 (7.9 to 18.3), 50 | 9.4 (6.1 to 13.6), 16 | **<0.0001** | **<0.0001** | >0.999 |
| **Z** | **0.6 (-0.8 to 2.4), 297** | 8.7 (4.5 to 12.6), 51 | 6.6 (4.2 to 11.0), 16 | **<0.0001** | **<0.0001** | >0.999 |
| **MRZ** | **0.2 (-0.9 to 3.0), 265** | 11.0 (8.4 to 15.3), 50 | 8.3 (5.9 to 11.0), 16 | **<0.0001** | **<0.0001** | >0.999 |
| **H** | 0.5 (-1.0 to 2.6), 157 | 1.9 (0.2 to 3.1), 17 | 5.3 (1.2 to 9.0), 16 | **0.0497** | 0.106 | 0.819 |
| **Δ Z Score Q_spec_ – Z score Q_IgG_, median (IQR), N** | | | | **p-values** | | |
| **M** | **-0.9 (-2.0 to 0.8), 289** | 6.2 (3.3 to 11.1), 51 | 5.1 (2.5 to 7.5), 16 | **<0.0001** | **<0.0001** | >0.999 |
| **R** | **-2.0 (-3.1 to 0.0), 279** | 6. 8 (3.6 to 10.5), 50 | 6.4 (4.0 to 12.4), 16 | **<0.0001** | **<0.0001** | >0.999 |
| **Z** | **-0.9 (-2.0 to 0.8), 297** | 2.3 (-0.2 to 6.0), 51 | 5.2 (0.6 to 6.9), 16 | **<0.0001** | **0.0005** | >0.999 |
| **MRZ** | **-0.6 (-1.7 to 0.6), 265** | 4.9 (3.6 to 8.3), 50 | 5.1 (4.3 to 6.9), 16 | **<0.0001** | **<0.0001** | >0.999 |
| **H** | 0.0 (-3.1 to 1.6),157 | **-4.1 (-5.1 to -0.8), 17** | 1.3 (-1.2 to 3.1), 6 | **0.027** | **0.038** | 0.407 |

PSIIR-: polyspecific intrathecal immune response negative, MS-S: MS spectrum, Non-MS CAIND: non-MS chronic inflammatory disease, IQR: interquartile range, AI: CSF/serum antibody index, M: measles, R: rubella, Z: Varicella zoster, H: Herpes simplex, MRZ: mean Z score of all patients with all three Z scores calculated. Q_spec_: pathogen-specific CSF/ serum IgG ratio, Q_IgG_: total CSF/ serum IgG ratio. N: number, IQR: interquartile range. Statistical analysis for differences across all three groups were performed using Kruskal-Wallis. Pair-wise statistical comparison was performed as Dunn’s post-test following Kruskal-Wallis test. Two-tailed p-values are given. Significant differences are indicated with bold letters.

**Supplementary Figures**

**
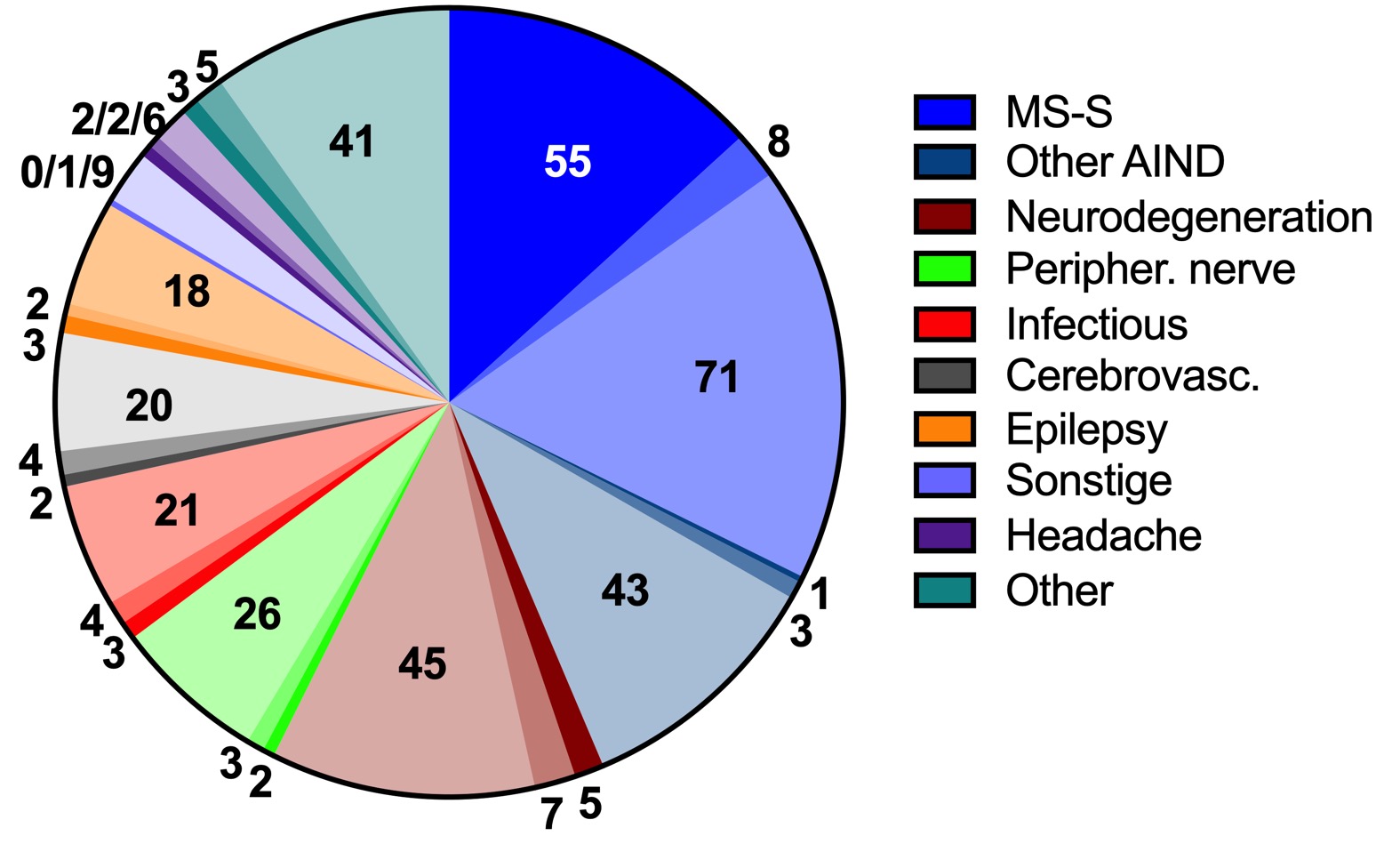
**

**Supplementary Figure 1 | Distribution of tentative diagnostic groups according to the case files in the 415 patients ≥ 50 years with positive OCB and polyspecific intrathecal immune response tested.** MS-S = MS spectrum diseases: all MS subtypes, clinically isolated syndrome, radiologically isolated syndrome. Other AIND = Other autoimmune-inflammatory neurological diseases, Neurodegenerative = Neurodegenerative diseases, Peripher. nerve = isolated peripheral or cranial nerve lesions, Infectious = infectious neurological diseases, Cerebrovasc. = cerebrovascular diseases, Other = other neurological diseases, PSIIR = polyspecific intrathecal immune response, PSIIR positive: darkest color, PSIIR definitely negative: lightest color, PSIIR unclear (to many antibody indices uncalculatable or increased due to CSF/plasma disequilibrium or infectious) in between. The numbers of patients are indicated (for percentages see Supplementary Table 1).

**
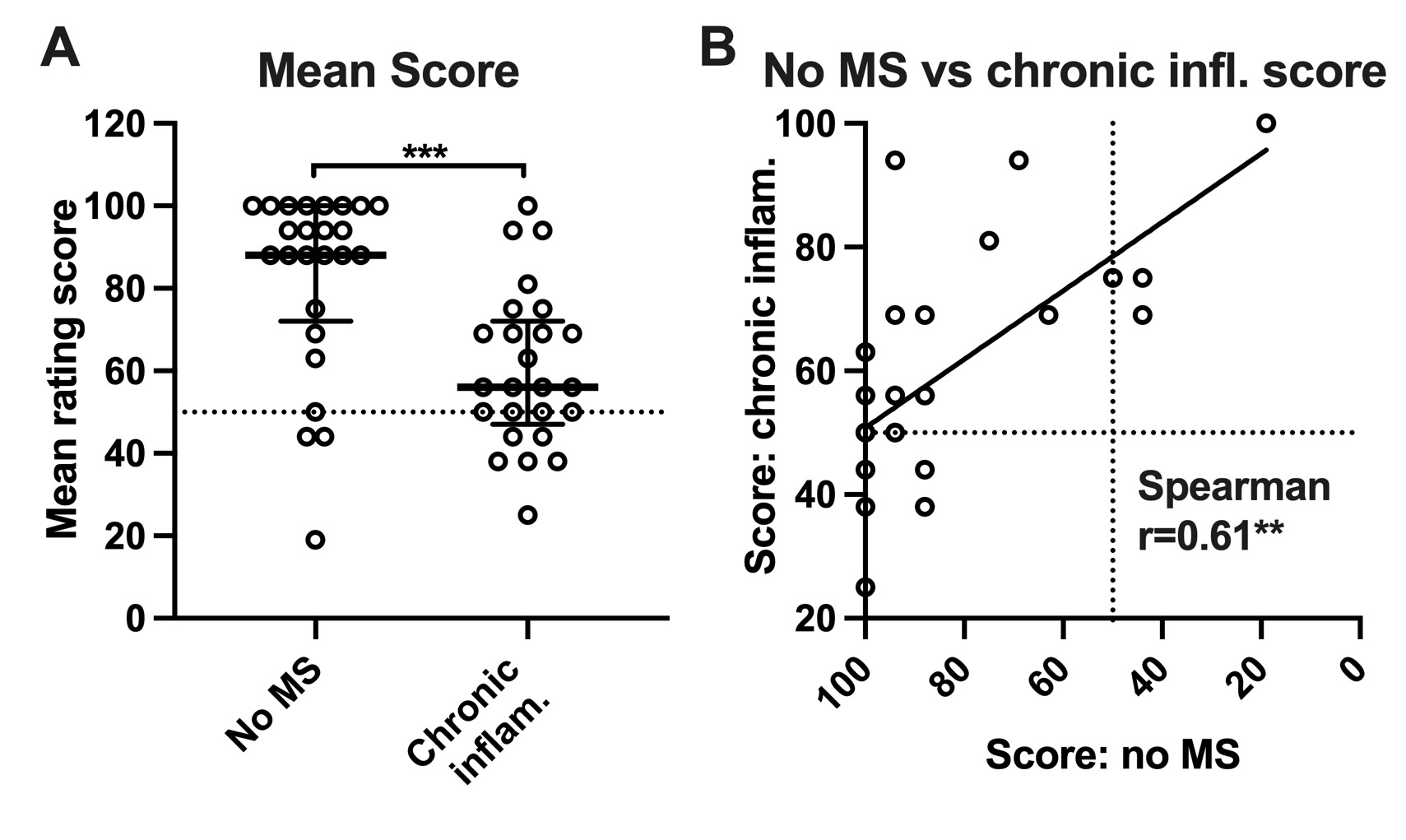
**

**Supplementary Figure 2 | Neuroimmunology expert rating of non-MS PSIIR-positive patients. (**A) The mean agreement rating scores for the statements “The patients does not suffer from MS (No MS)” and “The patient suffers from a chronic inflammatory disease of the nervous system (chronic. inflam.)” for all patients were compared. (B) The mean score for “chronic. Inflam.” was plotted againt the mean score for “No MS”. (C/D) The mean score of “No MS”. Some patient numbers are indicated (#). Statistical comparison ware performed using Mann-Whitney U tests (A) and Spearman rank correlation (B). The line in (B) indicates the results of a simple linear regress ion. **p<0.01, ***p<0.001.


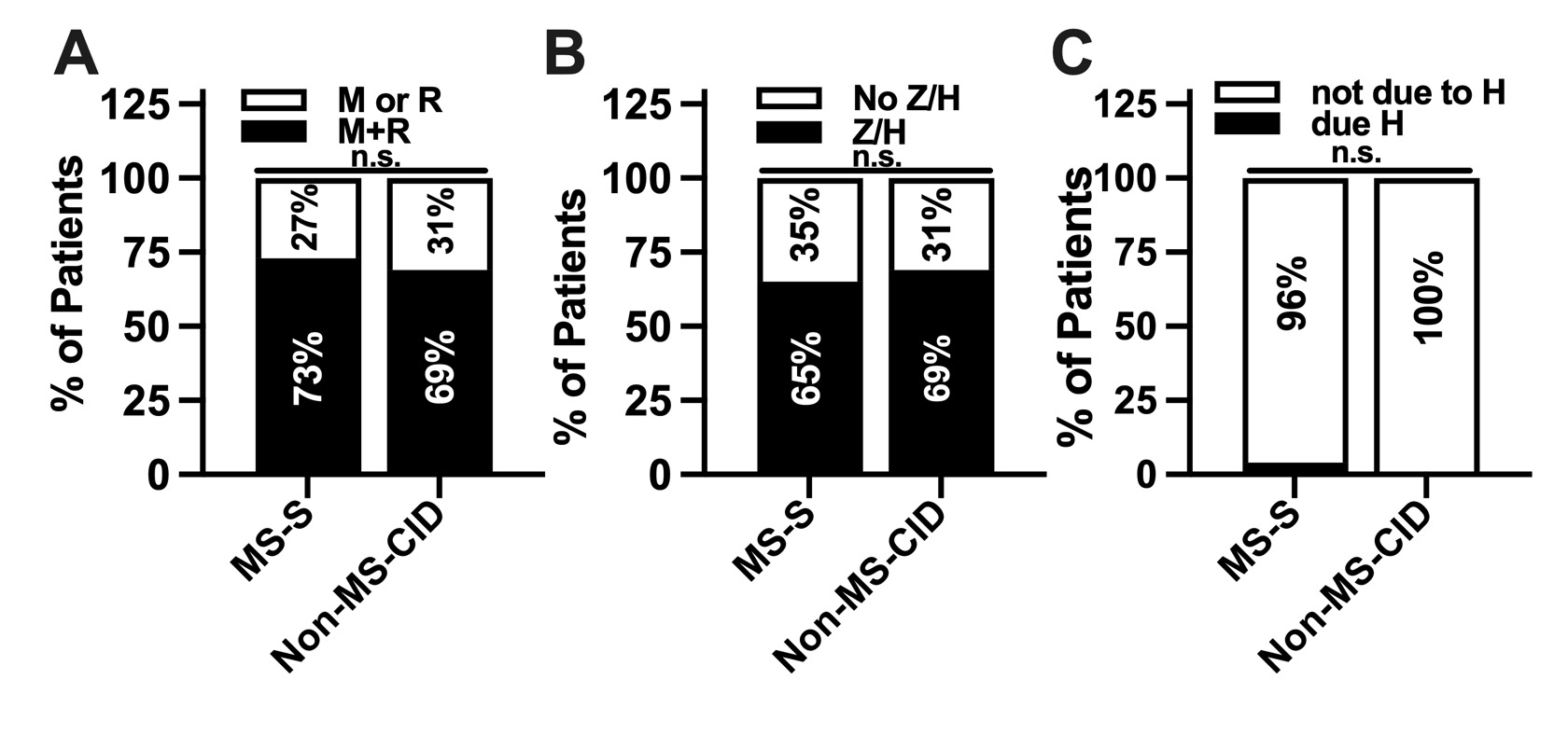


**Supplementary Figure 3 | Individual differences between certain characteristics of the PSIIR in MS-spectrum (MS-S) and non-MS chronic autoimmune-inflammatory disease (CAIND) patients.** The bar graphs show the proportion of patients in both groups with elevated AIs for (A) measles and rubella (M+R) compared to measles or rubella, (B) VZV and/or HSV (Z/H) or neither VZV nor HSV (no Z/H) as well as (C) the proportion of patients with a PSIIR rated positive bases on an elevated HSV-AI in combination with one elevated AI for either measles or rubella (due to H). Statistical comparison was performed using non-normalized data and Fisher’s exact tests, n.s. non-significant.

**Case Descriptions**

**Patients rated as unlikely to suffer from a chronic inflammatory neurological disease (Non-MS Non-CAIND)**

**Patient 1: Repeated tonic-clonic seizures possibly induced by hyponatremia**

After colon cleansing prior colonoscopy planned as a diagnostic procedure for unexplained abdominal pain, the 75-year-old female developed a tonic-clonic seizure followed by a prolonged disturbance of consciousness.

When transferred to our emergency room, the patient was disoriented. No focal neurological abnormalities were noted. While in the emergency room, she developed a second tonic-clonic seizure. Cerebral MRI showed mild periventricular and some juxtacortical white matter lesions (WML) suggestive of cerebral microangiopathy. Spinal MRI was not performed. Laboratory investigations showed hyponatremia (128 mmol/L, normal 132-154 mmol/L), hypochloridemia (95 mmol/L, normal 98-110 mmol/L). CSF analysis showed normal cell count (1 leukocyte/µl, upper normal limit 4 leukocytes/µl), normal blood-CSF barrier dysfunction (5.5 x 10^-3^, age-dependent upper normal limit 9.0 x 10^-3^), no quantitative intrathecal immunoglobulin synthesis but positive OCB restricted to the CSF. Measles AI and VZV AIs were elevated (3.0 and 1.5, respectively), rubella AI was normal (1.1). Follow-up plasma sodium was normal.

The patient fully recovered and was referred to a department for internal medicine to complete diagnostic work-up for abdominal pain. Levetiracetam was initiated as antiepileptic therapy, and an appointment in our epilepsy outpatient clinic was scheduled. However, the patient did not show up and was lost to follow-up.

**Patient 2: Unprovoked tonic-clonic seizure with chronic inflammatory CSF findings one year after Hanta virus infection**

This 51-year-old male was admitted after a bilateral tonic-clonic seizure, which had occurred during sleep. Directly after the episode, he had been unconscious for 15 min. When having regained consciousness, he reportedly had been disoriented. One year earlier, he had suffered from a severe Hanta virus infection with temporary kidney failure. His medical history did not reveal any other relevant information.

Upon neurological examination, he was awake and oriented, no abnormalities were detected. He showed a lateral tongue-bite. Cerebral MRI was normal. Spinal MRI was not performed. EEG showed intermittent right frontotemporal slowing. Compatible with the bilateral tonic-clonic seizure, plasma creatine kinase (CK) was highly elevated (3583 U/l, upper normal limit 171 U/l). CSF analysis showed normal cell count (2 leukocytes/µl, upper normal limit 4 leukocytes/µl), normal CSF-blood barrier function (Q_Alb_ 4.7 x 10^-3^, age-dependent upper normal limit 7.4 x 10^-3^). Lactate was normal (1.5 mmol/l, upper normal limit 2.6 mmol/l). OCB restricted to the CSF were positive. However, no evidence for quantitative intrathecal Ig synthesis was found. Measles and VZV AIs were elevated (1.7 and 3.7, respectively), rubella AI was normal (1.2).

An unprovoked epileptic seizure was diagnosed as the chronic inflammatory CSF findings were not judged to be causally related to the seizure. The patient was dismissed the next day. No follow-up information became available.

**Patient 3: Progressive cognitive impairment and asymptomatic cerebral ischemia**

The 83-year-old male was referred to our department for cerebrovascular diagnostic work-up of an asymptomatic subacute cerebellar ischemia, which had been detected upon cerebral MRI performed due to a 2-year-history of memory impairment.

Neurological examination revealed cognitive impairment compatible with dementia without any other neurological deficits (NIHSS: 0, mRS: 0). In addition to the subacute cerebral ischemia, the MRI showed prominent periventricular and subcortical white matter lesions compatible with cerebral microangiopathy. Spinal MRI was not performed. CSF showed normal leukocytes count (0 leukocytes /µl, upper normal limit 4 leukocytes/µl), no blood-CSF-barrier dysfunction (Q_Alb_ 5.3 x 10^-3^, age-adjusted upper normal <9.8 x 10^-3^), no evidence for quantitatively relevant intrathecal immunoglobulin synthesis but isolated OCB restricted to the CSF. Rubella (1.9), VZV (1.9) and HSV AIs (3.4) were elevated, measles AI was normal (1.1). Total Tau protein was elevated (824 pg/ml, <300 pg/ml) and Amyloid beta 1-42 was decreased (527 pg/ml, >550 pg/ml), compatible with Alzheimer’s disease pathology. Onconeural and antineuronal antibodies were negative. Ultrasound revealed severe atherosclerosis and mild stenosis of the left vertebral artery, which was considered causative for the cerebellar ischemia. Secondary prophylaxis with ASS and simvastatin was started.

At discharge, presentation to a memory clinic for neuropsychological testing was recommended. The patient was lost to follow-up.

**Patient 4: Ischemic stroke and mild cognitive impairment**

This 66-year-old female was admitted to the emergency department due to weakness of the right arm, which had first been noticed the day before admission. Additionally, the patient’s husband reported that his wife had appeared confused, e.g., wearing her glasses upside down. The previous week, she had been apathic spending most of the day in bed. Episodes of confusion had already appeared four months before admission. Upon admission to another hospital, these abnormalities had been interpreted as a side effect of chronic opioid intake. Opioid therapy had been initiated following radius fracture earlier that year and had led to opioid dependency. Neither patient’s history nor clinical examination revealed findings suggestive for connective tissue disease.

Neurological examination at admission showed mild aphasia, which limited assessment of other cognitive functions. In addition, mild central paresis of the right arm was noted. Cerebral MRI revealed subacute medial cerebral artery (MCA) ischemia in left temporo-parietal region. In addition, periventricular lesions compatible with cerebral microangiopathy and a mesial temporal atrophy were found. Spinal MRI was not performed. Beyond mild aphasia most likely explained by the MCA ischemia, neuropsychological examination performed ten days after admission showed deficits of orientation in time, mental processing speed, and impairment of executive functions. A duplex sonography revealed a high-grade left internal carotid artery (ICA) stenosis. EEG showed right frontotemporal slowing, but no epileptiform abnormalities. Blood tests revealed no relevant findings. Rheumatologic serology showed positive anti-histone and ds-DNA antibodies. CSF examination revealed a normal leukocyte count (1 leukocytes/µl, upper normal limit 4 leukocytes/µl) and normal blood-CSF barrier function (Q_Alb_ 4.9 x 10^-3^, age-dependent upper normal limit 8.4 x 10^-3^). OCB restricted to the CSF were associated with quantitative evidence for intrathecal IgG synthesis (11%). Measles, rubella as well as the VZV AI were elevated (4.8, 2.2, and 2.6, respectively).

In combination with the temporomesial atrophy and the localization of the cerebral , the cognitive impairments were judged to be pre-existent and not related to the cerebral ischemia. The ischemia was judged to originate from large-artery atherosclerosis. For ICA thromboendarterectomy, the patient was transferred to the department of vascular surgery. The patient did not present again to our department. Eleven years later, papillary renal cell carcinoma was diagnosed by our department of urology. At that time, no neurological symptoms were documented.

**Patient 5: Amyotrophic lateral sclerosis with chronic inflammatory CSF changes**

The 60-year-old male was admitted to our neurological ward for a second opinion after being diagnosed with ALS. However, diagnostic work-up two months earlier had indicated inflammatory CSF changes with positive OCB while CSF leukocyte count had been normal. Upon admission, the patient reported that 8 months earlier he had experienced progressive weakness of first his left hand and then the left arm. This had been associated with muscle wasting and twitching. Recently, his right hand had developed some weakness. He also reported that vertigo associated with nausea had developed at the time when he had first noticed the problems with his left hand. A quite similar episode with vertigo and nausea had occurred six years ago.

Upon neurological examination, no abnormalities of the cranial nerves were detected. The patient showed myatrophic distally pronounced paresis of the left arm as well as generalized fasciculations. In addition, finger extension on the right was slightly paretic. Deep tendon reflexes of the left arm were exaggerated. Sensory function was normal. Neuropsychological examination was normal. EMG demonstrated generalized acute and chronic neurogenic changes. Re-examination of recently performed cerebral and spinal MRI showed periventricular and juxtacortical WML while the spinal cord appeared normal. CSF analysis showed normal leukocyte count (2 leukocytes/µl, upper normal limit), slight blood-CSF-barrier dysfunction (Q_Alb_ 11.4 x 10^-3^, age-dependent upper normal limit <8.0 x 10^-3^) and isolated OCB restricted to the CSF without any evidence of quantitative intrathecal Ig synthesis. CSF lactate was normal (1.6 mmol/L). Measles and VZV AIs were elevated (2.4 and 2.2, respectively) while rubella AI was normal (0.9). Rheumatological serology was normal. General laboratory examination showed slight leukocytosis (11.2 Giga/L, normal 4.3 -10.8 Gigal/L). CRP was slightly elevated (8.0 mg/L, normal <5 mg/L). As the clinical picture was compatible, amyotrophic lateral sclerosis was diagnosed despite the inflammatory CSF findings.

Upon follow-up examination as an out-patient 13 months later, the patient reported that weakness of his hands had massively progressed. In addition, leg weakness had developed so that walking without help had become difficult. Still, no difficulties in speaking or swallowing were reported. Due to daytime sleepiness, non-invasive ventilation had been prescribed in the meantime that he would use 7 hours per night. Neurological examination showed slight tongue hypomotility but no tongue fibrillations or atrophy. No other cranial nerve abnormalities were found. He showed myatrophic tetraparesis, severe of the left arm, moderate of the right arm, mild to moderate of the legs. Deep tendon reflexes were increased, plantar responses were flexor bilaterally. Generalized fasciculations were present. Another 10 months later, the muscle weakness again had progressed. The arms had become almost plegic. He reported that he had become wheelchair-bound several months ago. He reported difficulties to hold his head upwards. Speech had become low. Sometimes he would choke while drinking, but not when eating. He had lost 10 kg of body weight. Neurological examination then showed tongue fibrillation, atrophy and hypomotility. Palmomental reflex was positive bilaterally. In addition, there was a severe myatrophic tetraparesis with almost plegic arms and exaggerated reflexes. Plantar response remained negative bilaterally. No sensory disturbances were noted. As the clinical course was still compatible with the diagnosis, no second LP was performed. The patient was lost to follow-up after this outpatient appointment 23 months after first admittance.

**Patient 6: Probable bulbar-onset motor neuron disease**

An 82-year-old male patient presented because of progressive dysphagia that had been present for one year. Clinically, the patient presented with dysarthria and dysphagia in the sense of bulbar symptoms with tongue paresis as well as increased reflexes in the upper extremities. Cerebral MRI showed no explanatory pathology but few periventricular WML. Spinal MRI did not show any spinal cord lesions. EMG revealed acute and chronic neurogenic changes in the genioglossus muscle, but not in the arm and leg muscles. CSF analysis showed normal cell count 1/μl (<5/μl), normal blood-CSF barrier function (Q_Alb_ 7.0 x 10^-3^, upper normal limit 9.5 x10^-3^), normal lactate (1.8 mmol/l), but OCB isolated in the CSF without quantitative evidence of intrathecal IgG, IgA or IgM synthesis. The rubella AI was slightly elevated (1.5,) as was the HSV AI (1.7), while measles and VZV AIs (1.4 and 1.0 respectively) were normal. CSF pNFH was highly elevated (5619 pg/ml, cut-off for MND > 560 pg/ml). The diagnosis of bulbar MND was made. No follow-up data of this patient are available.

**Patients rated to probably suffer from a non-MS chronic-inflammatory disease of the nervous system**

**Patient 7: Provoked repetitive seizures associated pneumonia and ciprofloxacin treatment**

The 86-year-old female was admitted to our department after she had been found non-responsive with muscle twitches of her left arm by the staff of the patient’s nursing home. She had been known to be cognitively impaired and unable to walk after repeated osteoporotic fractures of the lumbar dorsal spine. During the emergency transport, two bilateral tonic-clonic seizures had occurred so that the patient had received 3 mg of midazolam. Three days before admission, antibiotic treatment with ciprofloxacin for urinary tract infection had been initiated.

Upon hospital admission, the patient was soporose, but slowly regained consciousness. Lung auscultation was suggestive for pneumonia. Blood tests revealed a leukocytosis of 20 G/L (normal 4.3 -10.8 G/L) and an elevated CRP of 32 mg/L (normal <5 mg/L). Cerebral MRI showed moderate to severe subcortical and periventricular WML suggestive for cerebral microangiopathy. Spinal MRI was not performed. As emergency CSF parameters with normal cell count (1 leukocyte/µl, upper normal limit 4 leukocytes/µl) and CSF lactate (3.8 mmol/L, normal 1.7–2.6 mmol/l) increased in parallel with CSF glucose (4.5 mmol/L, normal 2.7–4-2 mmol/L) ruled out meningitis, the patient was transferred to another hospital’s department of internal medicine for treatment of pneumonia. The seizures were judged as provoked by both systemic infection and ciprofloxacin. The full CSF results available after the patient had been transferred showed normal CSF-blood barrier function (Q_Alb_ 7.1 x 10^-3^, upper normal limit 9.7 x 10^-3^) and positive OCB restricted to the CSF not associated with quantitative intrathecal IgG, IgA and IgM synthesis. Rubella, varicella as well as measles AIs all were elevated (1.7, 1.6, and 1.7, respectively).

The positive OCBs and elevated AIs were judged as signs of a clinically silent inflammatory CNS process of chronic autoimmune origin. The patient was lost to follow-up.

**Patient 8: Fluctuating vigilance and depressive mood with suicidal ideation**

The 68-year-old male was admitted to the emergency room and transferred to the department of neurology one day later due to a worsening of general condition and fluctuating consciousness. His medical history included coronary and peripheral artery disease, chronic kidney failure, chronic obstructive pulmonary disease, scoliosis with chronic lumbalgias treated with opioids, recurrent sinusitis as well as polyneuropathy, symptomatic epilepsy and reportedly more than 30 episodes of viral meningitis.

At the time of admittance to the emergency room, psychomotor slowing was observed. Urinary tract infection was diagnosed and treated with antibiotics (ampicillin/sulbactam). Neurological examination revealed dysarthria and gaze-evoked nystagmus. After referral to the department of neurology the next day, the patient complained of chronic headaches, which had reportedly been present for years but had been exacerbated after reduction of fentanyl transdermal patch from 100 to 50µg/h a couple of days ago. In addition to atactic gait, severe depression with acute suicidal ideation became apparent. Cerebral MRI only showed periventricular and juxtacortical WMLs best compatible with cerebral microangiopathy. Spinal MRI performed 11 months earlier had not shown any signal abnormalities in the spinal cord. General laboratory examination revealed leukocytosis (13.5 Giga/L, normal 4.3 -10.8 Gigal/L) and elevated CRP (157.0 mg/L, normal <5 mg/L). CSF showed a pleocytosis (18 leukocytes/μl, upper normal limit 4 leukocytes/µl) without blood-CSF barrier dysfunction (Q_Alb_ 3.9 x 10^-3^, age-adjusted upper normal <8.5 x 10^-3^) but positive oligoclonal bands (OCB) restricted to the CSF as well as quantitative intrathecal IgM synthesis of 37% (normal 0). Rubella (4.9), VZV (1.9) and HSV AIs (3.0), were elevated, the measles AI was normal (1.0). Blood levels of his anti-epileptic medication, valproate and eslicarbazepine as well as its metabolites were in normal therapeutic range.

Due to acute suicidal ideation, the patient was referred to the department of psychiatry of a neighboring hospital. Here, repeated episodes of disorientation and reduced consciousness occurred. Due to these unexplained episodes, the patient was transferred to the neurological intensive care unit of the neighboring hospital. As the final discharge report was not sent to our department, the final diagnosis and outcome remains unknown.

**Patient 9: Diverse unexplained symptoms of somatoform etiology**

The 52-year-old male presented to the emergency room due to fluctuating paresthesias of both feet and palms starting 4-5 months ago. Intermittently, these paresthesias would exacerbate and extend up to his knees. These episodes reportedly occurred 5-10 times per month, last 30-60 minutes and were associated with deep pain in the legs. He also reported to suffer from sudden sleep attacks that would occur independently of the body position up to 10 times daily. When standing, he would fall. The falls had led to several minor injuries. However, he reported to never have experienced tongue biting, enuresis or muscle pains associated with these attacks. He reported to have problems to remember where he was when regaining consciousness after these attacks. He also reported amnestic episodes that might have lasted hours as he had driven his car over long distances during these episodes. He also reported disturbed sleep for years, but no general daytime sleepiness. Due to chronic lumbalgia, he had been on sick leave for three months. Although his general practitioner had prescribed tetrazepam and tramadol, he wouldn’t take these medications on a regular basis. Until four months before admittance, he had been self-employed but then was forced to file bankruptcy. The employment he got afterwards had only lasted for one month. Due to repeated left thoracal pain, he had undergone cardiologic examination including electro- and echocardiography two months before admission, which yielded unremarkable results. One month later, he had been seen by a neurologist where he had reported lumbalgias with pain radiating into the right leg as well as sudden weakness of both leg with falls without loss of consciousness. Lumbal MRI one month before admission had shown degenerative changes without relevant spinal or neuroforaminal stenoses but pseudospondylolisthesis grade 1 L3/4.

Upon admission in our department, neurological examination did not reveal any reproducible abnormalities or obvious cognitive deficits. MRI showed minor subcortical gliotic changes. Spinal MRI was not performed. Nerve conduction studies (NCS) and electroencephalography (EEG) were normal. Quantitative sensory testing did not reveal a sensory deficit. A Schellong test as well as 24 hour-ECG were normal. Onconeuronal and rheumatological serologies were negative. Cerebrospinal fluid analysis showed a normal cell count (0 leukocytes/µl, upper normal limit 4 leukocytes/µl), slightly disturbed CSF-blood barrier function (Q_Alb_ 11.4 x 10^-3^, age-dependent upper normal limit 7.4 x 10^-3^) and positive OCB restricted to the CSF. No quantitative intrathecal IgG, IgA or IgM synthesis was detected. Measles AI was 2.3, rubella AI was 2.6 and VZV AI was 3.4. Hypocretin-1 in CSF was 326 pg/ml (normal >110 pg/ml).

In summary no relevant neurological abnormalities that explained the diverse symptoms reported by the patient could be identified although chronic inflammatory changes were found in the CSF. The multiple different symptoms that also were reported quite differently to the physicians the patients consulted were best explained as somatoform as a result of a depressive episode. The patient was lost to follow-up.

**Patient 10: Progressive mood changes, cognitive impairment, dysarthria and left-sided spastic paresis**

The 62-year-old male was admitted for a second opinion regarding still ill explained, slowly progressive combination of neurological deficits with slurred speech, spastic paresis of the left leg associated with depressed mood and cognitive deficits, which had started to develop almost two years earlier. Eighteen months prior admission to our department, a cerebral MRI had only shown one prominent periventricular WML adjacent to the left lateral ventricle and few additional smaller periventricular and subcortical lesions. In addition, a global atrophy had been observed. Spinal MRI had revealed multiple disc protrusions on the cervical, thoracic, and lumbosacral level, but no spinal cord abnormalities were documented. Six months later, he was admitted to another department of neurology for diagnostic workup after right facial weakness had developed several days earlier. At that time, he reported that two months earlier after hernia surgery he had noted some weakness of his left leg. Upon neurological examination, central facial paresis on the right and central paresis of the left leg with extensor plantar response were noted. Neuropsychological examination had revealed executive dysfunction, verbal working memory, semantic and phonematic verbal fluency had been impaired. Somatosensory evoked potentials showed prolonged latencies. CSF analysis had shown normal leukocyte count (1 leukocyte/µl, upper normal limit 4 leukocytes/µl), slightly impaired blood-CSF barrier function (Q_Alb_ 8.6 x 10^-3^, age-adjusted upper normal limit 8.1 x 10^-3^), positive OCB restricted to the CSF without evidence of quantitative intrathecal Ig synthesis, slightly elevated total Tau protein (310 pg/ml, upper normal limit 300 pg/ml), normal amyloid beta 1-42 (1283 pg/ml, lower normal limit 550 pg/ml). The measles AI had been found elevated to 2.1, the rubella AI to 2.1 and the VZV AI to 1.5. At re-admission 5 months later, he reported that the dysarthria had progressed as well as the gait instability. Transcranial motor evoked potential had been normal as were NCS and EMG. CSF analysis upon repeat lumbar puncture was unchanged (measles AI 2.0, rubella AI 1.5, VZV AI 1.9). In addition, vitamin B12 deficiency was diagnosed and substitution advised. Upon psychiatric admission four months later, it was documented that the gait disturbance again had become worse. After a first episode of depression 4 years earlier, he had become increasingly anxious, depressed, and lacked energy. Thus, antidepressant medication had been started before admission without relevant effect. This wife reported that his personality had changed. Neurological examination showed spasticity of the left hand and leg as well as dysarthria. Repeat-MRI did not reveal any new findings. EEG was normal. FDG-PET showed a left frontal and right temporal decrease in glucose utilization. CSF showed mild CSF-blood barrier dysfunction, positive OCB and a measles AI of 2.2, rubella AI of 2.3, and VZV AI of 1.2. Frontotemporal lobar degeneration with central neuron degeneration was mentioned as a differential diagnosis and the patient was referred to our department, where he was first seen as an out- and then as an inpatient 13 months after initial admission to a department of neurology.

In addition to the known complaints, he then reported that his left hand would sometimes develop painless cramps and his finger would stay in an odd position. Sensory dysfunction was denied. In response to physical activity, he had recently developed some shortness of breath. Although he reported not to have any problems with memory or concentration, his wife reported the impression that his memory had declined. In addition, she had noted some wordfinding difficulties. Reportedly, an atrophic gastritis had been diagnosed as cause for vitamin B12 deficiency.

Upon neurological examination, dysarthria as well as reduced tongue motility were noted. The left leg was slightly paretic with increased reflexes of both legs and an extensor plantar response of the left foot. There was dysdiadochokinesis as well as impaired fine motor skills of the left hand. Neuropsychological examination revealed executive dysfunction, impairment of mental processing speed as well as non-verbal episodic memory. Cerebral MRI was unchanged WML. Global atrophy appeared slightly more pronounced on the right hemisphere. Spinal MRI was not performed. NSC did not reveal any relevant abnormalities. EMG reveals signs of acute denervation in the right lateral vastus muscle only. Oculomotor analysis showed hypometric saccades and decrease saccade velocity. CSF analysis showed normal leukocyte count (0 leukocytes/µl), slightly perturbed blood-CSF barrier dysfunction (Q_Alb_ 8.8 x 10^-3^, age-dependent upper normal limit 8.2 x 10^-3^). Lactate was normal (2.2 mmol/l, upper normal limit 2.6 mmol/l). OCB restricted to the CSF again were positive. However, no evidence for quantitative intrathecal Ig synthesis was found. Measles AI was 2.0, rubella AI was 2.0 and a VZV AI was 1.1. Onconeuronal and antineuronal antibodies were negative. Total Tau protein was borderline (310 pg/ml, upper normal limit 300 pg/ml), amyloid beta 1-42 was normal (1043 pg/ml, lower normal limit 550 pg/ml). Due to the left-sided lateralization of motor deficits a disease related to cortico-basal degeneration was assumed. The patient was lost to follow-up.

**Patient 11: Headache with dural enhancement**

The 54-year-old male was referred from the ENT department where he presented with persisting bifrontal headache starting 5 days ago. CT had excluded acute sinusitis but showed swelling of the mucous membranes of both maxillar sinuses.

Upon neurological examination, no abnormalities were found. Cerebral MRI revealed no parenchymal abnormalities. However, dural thickening and contrast enhancement was noted. Spinal MRI was not performed. Laboratory investigations showed highly elevated C-reactive protein (CRP 145 mg/L, normal <5 mg/L). CSF showed mild pleocytosis (6 leukocytes/µl, upper normal limit 4 leukocytes/µ), no blood-CSF-barrier dysfunction (Q_Alb_ 3.4 x 10^-3^, age-dependent upper normal limit 7.6 x 10^-3^), positive oligoclonal bands (OCB) restricted to the CSF as well as quantitative intrathecal IgG synthesis of 19% (normal 0%). Measles and VZV AIs were elevated (2.4 and 4.9, respectively, normal <1.5), rubella AI was normal (1.2). An antibiotic therapy with ceftriaxone was initiated, which within 7 days was associated with a slight decrease in CRP to 131 mg/L upon dismissal.

The patient did not show up for a scheduled follow-up appointment in our outpatient clinic and thus was lost to follow-up.

**Patient 12: Headache and hearing loss**

The 63-year-old male reported that he had developed frontal headache two weeks before hospital admission. After diagnosis of frontal sinusitis and prescription of antibiotics, he developed rash, but the headache persisted. He was admitted for diagnostic work-up suspected meningitis after having developed recurrent fever.

Upon admission, the neurological examination did not reveal any abnormalities. Cerebral MRI did not show any relevant abnormalities but frontal sinusitis. Spinal MRI was not performed. CSF showed 49 leukocytes/µl (upper normal limit 4 leukocytes/µl), slight blood-CSF barrier dysfunction (Q_Alb_ 10.4 x 10^-3^, age-dependent upper normal limit 8.2 x 10^-3^), no OCBs and slightly elevated AIs for VZV (1.8) and HSV (1.5). Measles AI (1.0) and rubella AI (0.9) were normal. The patient was treated empirically with intravenous aciclovir and ceftriaxone. Several days later, he developed bilateral sensorineural hearing loss and hypoglossal palsy on the left. Repeat LP 9 days after initial CSF analysis showed slightly decreased CSF cell count (35 leukocytes/µl), slightly less blood-CSF barrier function (9.4 x 10^-3^), positive OCBs in CSF without evidence for quantitative intrathecal immunoglobulin synthesis and unchanged AIs (Measles 0.9, Rubella 1.0, VZV 1.8, HSV 1.5). While headache improved, last LP before dismissal twenty days after initial LP revealed a somewhat lower cell count (17 leukocytes/µl), normalized blood-CSF barrier function (Q_Alb_ 7.7 x 10^-3^), now associated with a positive MRZ reaction (VZV AI 1.8, HSV AI 1.0, measles AI 0.7, rubella AI 4.0).

Fourteen years later, the patient again was admitted due to an additional episode of neurological symptoms. In addition to the still persistent hypoacusis from the first episode, he reported hoarseness and swallowing difficulties with globe feeling starting one month earlier and sudden hearing loss on the left ear the day before admission. In addition, he reported repeated vertigo and facial hypaesthesia on the left. cMRI did not reveal any relevant abnormalities. ENT examination did not find any explanations for the reported globe feeling. No hypoglossal palsy was noted. However, fiberoptic endoscopic evaluation of swallowing revealed penetration and aspiration. CSF analysis showed normal cell count (2 leukocytes/µl), normal CSF-blood barrier function (Q_Alb_ 8.1 x 10^-3^), OCB were positive in the CSF, measles AI was 0.7, rubella 1.0, VZV 1.0 and HSV 1.5.

**Patient 13: Visual disturbances, peroneal axonal lesion and possibly central pallhypaesthesia**

Upon admission, this 66-year-old female reported blurred vision on her left eye starting two weeks earlier. In addition, she reported of continuous headache starting two weeks ago, which had considerably worsened within the last three days before admission. The patient did not spontaneously report any other complaints suggesting neurological dysfunction.

Upon neurological examination, visual acuity was reduced to 0.6 on the left eye. Ankle jerk reflexes were reduced bilaterally. There were distally pronounced pallhypaesthesias without any hypaesthesia for touch or temperature. Romberg’s test was positive supporting impaired proprioception. Cerebral MRI showed multiple subcortical and juxtacortical WML. Spinal MRI was not performed. NCS showed an axonal lesion of the right peroneal nerve. Correspondingly, acute as well as chronic neurogenic change of the right anterior tibial muscle were noted. Tibial sensory evoked potentials were normal as were visual evoked potentials. Retinal fluorescence angiography did not show any abnormalities. Rheumatological serology as well as CRP were normal. Blood sedimentation rate was only marginally increased (30 mm/h). Temporal artery biopsy was declined by the patient. CSF analysis revealed normal cell count (1/µl), blood-CSF-barrier function was normal (3.8 x 10^-3^, upper normal value 8.4 x 10^-3^), OCB restricted to the CSF were present. Reiber formulas indicated quantitative intrathecal IgG synthesis (17%), but not evidence for intrathecal IgA and IgM synthesis was found. AI for measles, rubella, VZV and HSV all were elevated (1.6, 2.6, 4.5, and 1.8, respectively).

Oral prednisolone (100 mg) was initiated upon which the subjective disturbance of vision completely resolved. In combination with headache and normal VEP a vasculitic anterior ischemic optic neuropathy was assumed to be the most likely diagnosis. The patient was lost to follow-up.

**Patient 14: Vertigo associated with otitis media**

The 80-year-old male was admitted with dizziness, nausea and worsening of a pre-existing gait disturbance due to a pre-existing peripheral neuropathy. Following otitis media on the right, a tympanostomy had been performed two weeks before.

Dix-Hallpike manouver to the left upon admission revealed a rotatory nystagmus to the left. In addition, a spontaneous nystagmus to the right side was observed. The patellar tendon reflexes were reduced bilaterally, the ankle jerk reflexes were absent. The radiological report of the cerebral MRI performed upon admission did not mention any parenchymal abnormalities but opacification of the mastoid air cells on the right. A spinal MRI was not performed. CSF analysis showed normal leukocyte count (1 leukocytes/µl, upper normal limit 4 leukocytes/µl), no blood-CSF-barrier dysfunction (Q_Alb_ 6.0 x 10^-3^, normal 9.4 x 10^-3^), no evidence for quantitative intrathecal immunoglobulin synthesis but positive OCB restricted to the CSF. Measles (1.6), rubella (1.5) and VZV (1.8) AIs were elevated. In summary, the symptoms were rated as vestibular neuritis. As possible differential diagnosis, an inflammatory vestibular involvement as part of the otitis media was discussed. After consultation of the ENT department, an antibiotic therapy with cefuroxime (1,5 g/d for 10 days) as well as an anti-inflammatory therapy with prednisone (250mg/d for 3 days and slow tapering) were started.

Afterwards, the patient was referred to ENT department for further diagnostic and therapy. In the final report, a CT of the petrous bone was inconspicuous, and the patient was discharged home fully recovered. Due to lumbalgias, a MRI of the lumbar spine was performed which in not show any lesion in the lower thoracal spinal cord and the conus and severe arthrotic changes of lumbal spinal with *de novo* scoliosis.

Two years later, he was again admitted due to an episode with disorientation, slurred speech and weakness of the right extremities. Cerebral MRI did not show moderate periventricular WML compatible with cerebral microangiopathy. EEG was normal. Repeat lumbar puncture now did not show any isolated OCB. A symptomatic epileptic seizure due to cerebral microangiopathy was diagnosed and anti-epileptic medication with lamotrigine recommended. A cervical MRI performed due to cervicalgias did not show any spinal cord lesions of the cervical and upper thoracic cord.

Nine years later, the patient was re-admitted due of an acute visual impairment of the right eye, headache, jaw claudication and pulsation of the temporal artery. CRP and erythrocyte sedimentation rate (60mm/h) were elevated. The biopsy of the temporal artery revealed chronic and florid inflammation of the tunica media, compatible with a giant cell arteritis although no giant cells were detected. Rheumatologic serology showed borderline ANAs (1:80) with positive PMSCL antibodies. Symptoms rapidly improved after initiation with high-dose methylprednisolone (500 mg intravenously) following oral prednisolone.

**Patient 15: Possibly central paresis of finger extension associated with CREST syndrome**

Upon admission to our department of neurology, the 78-year-old female reported weakness of her right little finger associated impairment to extent the little finger, which had slowly progressed within the last six months. Seven years ago, she had been diagnosed CREST syndrome with increased ANA and centromere antibodies and lymphocytopenia associated with slight esophageal dysmotility, after she had developed eczema after sun exposure as well as slight dysphagia with sometimes slight discomfort and pain while swallowing. She also had reported to the rheumatologist to suffer from Raynaud’s syndrome with painful white fingers upon cold exposure for decades. At that time, no specific immunomodulatory treatment had been initiated. Reportedly, repeated episodes of thrombocytopenia of unknown origin had occurred for decades.

At admission to our department, neurological examination revealed paresis of finger extension, pronounced for the little finger, as well as paresis of little finger abduction on the right. Ankle jerks were slightly asymmetric (left > right). Slight pallhypaesthesia as well as minor disequilibrium upon tandem walking were noted. Cerebral MRI only showed minor periventricular WML, most likely of microangiopathic origin. The cervical spinal cord MRI did not show any lesion. Neither neuroforaminal stenoses nor disc herniations explanatory for the neurological deficits were found. NCS were normal. EMG did not any unequivocal abnormalities. X-ray of the right hand did not show any arthropathic changes. CSF analysis showed normal leukocyte count (3 leukocytes/µL), normal blood-CSF barrier function (Q_Alb_ 6.5 x 10^-3^, upper normal limit 9.2 x 10^-3^), lactate was normal (1.4 mmol/L). Positive OCB were associated with quantitative intrathecal IgG and IgA synthesis (41 and 47%, respectively). AI for measles, rubella, VZV and HSV were increased (4.5, 1.9, 6.0, and 1.9, respectively). Onconeuronal antibodies were negative. ESR was increased (75 mm/h). Platelets were normal. Rheumatologic serology again showed ANAs (>1:2560, upper normal limit 1:80) with positive centromere antibodies (>240 U/ml, upper normal limit 10 U/ml).

Taken together, central paresis of the right hand associated with intrathecal inflammation associated with the CREST syndrome was assumed. The hematological advice was to start corticosteroid in combination with methotrexate or hydroxychloroquine. The patient was subsequently lost to follow-up.

**Patient 16: Progressive spastic tetraparesis with sensory disturbances**

Upon admission to our department, the 58-year-old male reported a slowly progressive gait disturbance starting 1 year before. Initially, he had observed reduced sweating of his feet. In addition, numbness, paraesthesias and burning sensations of the right foot had started, then the left foot had followed. Upon admission, he reported that the fluctuating painful paraesthesias had started to radiate into his entire legs. Walking would only be possible with a walker.

Neurological examination revealed spastic tetraparesis with exaggerated reflexes and bilateral extensor plantar responses. The patient reported hypaesthesias for touch of the entire legs and both hands, distal pallhypaesthesia (4/8). In addition, thermhypaesthesia of the right leg was found. Gait was spastic atactic. Cerebral MRI showed very few subcortical WML. Spinal MRI did not show any lesions. CSF analysis showed pleocytosis (10 leukocytes/µl, upper normal limit 4 leukocytes/µl), very minor blood-CSF barrier dysfunction (Q_Alb_ 8.0 x 10^-3^, upper age-adjusted normal limit 7.9 x 10^-3^) and positive OCB with 39% intrathecal IgG synthesis. Lactate was normal (1.8 mmol/l, upper normal limit 2,6 mmol/l). Measles and rubella AIs were elevated (3.6 and 4.9, respectively). VZV and HSV AIs were normal (1.0 and 0.6, respectively). Rheumatologic serology was normal, aquaporin 4 antibodies were negative.

A course of high-dose methylprednisolone was administered which led to marked but transient improvement in his ability to walk, which transiently was without aid. Seven months later urinary urge was reported. Ten months after corticosteroid treatment, he reported by be permanently dependent on a walker, later in the day even a wheelchair. He also reported to suffer from urge incontinence. Neurological examination revealed an asymmetric spastic tetraparesis, more pronounced on the left, with extensor plantar responses. Again, there was hypaesthesia for touch and temperature of both legs more pronounced on the right and bimalleolar pallhypaesthesia (4/8). Plantar reflex was extensor bilaterally. Cerebral and spinal MRI did not show any lesions suggestive for an inflammatory CNS disease. TMS showed an increased central motor conduction time to both legs. NCS were normal. Vitamin status was normal with exception of a slight vitamin B6 deficiency. Repeat CSF analysis showed a normal cell leukocyte (1/µl), blood-CSF-barrier function was normal (Q_Alb_ 8.1 x 10^-3^, age-dependent upper normal limit 8.1 x 10^-3^), no quantitatively relevant intrathecal immunoglobulin synthesis was detected, OCB in CSF were positive. Measles and Rubella AIs were elevated to 2.5 and 5.7, respectively, while VZV AI was normal. Again, high-dose methylprednisolone (1g for 5 days) was initiated, followed by oral prednisolone, for which slow tapering to 7.5 mg was suggested after dismissal. When seen as an outpatient, he reported that the corticosteroid therapy, currently 10 mg oral prednisolone, had not led to any improvement of his condition. Two years after initial admission to our department, neurologic work-up was repeated in another hospital due to progression of his gait disturbance. Reportedly cerebral MRI showed unspecific supratentorial WMLs, spinal MRI did not reveal any lesions. Tibial SEP were absent, TMS again showed pyramidal tract lesion. Again, high-dose intravenous corticosteroids were given, 10 mg methotrexate 1/week were initiated. Three years after initial presentation, the patient presented again to our outpatient clinic. He reported that the last corticosteroid treatment had again led to transient improvement. Thus, oral prednisolone was advised. He was again seen as an outpatient 4.5 years after initial admission in our department. He reported to have become wheelchair-dependent. As oral prednisolone, which he had taken at a dose of 100 mg for two months, had not led to any beneficial effects, he was advised to taper the dose. A new admission for repeat diagnostic work-up was suggested, which the patients declined.

**Patient 17: Progressive spastic tetraparesis and intermittent neuropathic pain**

The 50-year-old male was first admitted to our department for further diagnostic work-up of progressive weakness of both legs associated with intermittent pain and numbness of both legs. About a year prior admission, he reportedly had developed weakness of the left leg of rather sudden onset. The weakness had progressed to foot and toe extension. Two months later also the right leg had become weak. When longer in a sitting position, his left leg would be become numb. He intermittently experienced radiating pain in the medial ventral right thigh. At the time of admission, he reported to suffer from generalized muscle twitching and cramps, especially in the right calf. Prior diagnostic work-up in several other departments of neurology had excluded stiff-person-syndrome. Due to a positive MRZ reaction found in CSF a chronic-inflammatory disease had been diagnosed. Corticosteroids and plasma exchange had not resulted in any clinical improvement. Two years prior to admission, a medullary thyroid carcinoma had been detected and treated with thyroidectomy.

Upon admission, neurological examination revealed spasticity in both legs with foot and toe extension and flexion paresis on the left. Deep tendon reflexes of the arms were normal but pathologically increased in both legs. Plantar responses were extensor bilaterally. Somatosensory function was normal. Some muscle wasting of the left thenar, the right thigh and both calves was noted. Somatosensory function was normal with exception of a slight bimalleolar pallhypaesthesia (6/8). Cerebral and spinal MRI did not reveal any abnormalities. NCS showed reduces amplitudes of all sensory nerves. EMG showed signs of acute denervation in the left abductor pollicis brevis muscle only but not widespread signs of acute or chronic denervation. CSF examination showed a normal leukocyte count (0 leukocytes/µl, upper normal limit 4 leukocytes/µl), no blood-CSF-barrier dysfunction (Q_Alb_ 6.7 x 10^-3^, upper normal limit 7.3 x 10^-3^), no evidence for quantitatively relevant intrathecal immunoglobulin synthesis but positive OCB. Measles and rubella AI were elevated (3.9 and 1.6, respectively), VZV AI was normal. Onconeuronal and aquaporin 4 antibodies were negative. Rheumatological serology did not reveal any abnormalities. Although neuroimaging did not disclose any demyelinating lesions, a disease closely related to primary progressive multiple sclerosis was assumed. The patient was treated with mitoxantrone in another hospital after dismissal. Here, progressive atrophy of the left thenar and the right thigh was noted. In combination with generalized fasciculations, a motor neuron disease was suspected. The patient was re-admitted to our department for a second opinion nine months after the initial admission. Neurological examination now showed an incomplete left oculomotor and right hypoglossal palsy. There was a left thenar and right quadriceps atrophy. Elbow extension on the right was slightly paretic as was finger abduction bilaterally. Foot extension and flexion was paretic on the left. Again, spastic paraparesis was noted. Repeat NCS again did not reveal widespread acute and chronic denervation typical for MND, rather myopathic MUPs were found in some muscles. Repeat LP again showed normal cell count (2 cells/µl), OCB restricted to the CSF and a positive MRZ reaction (measles AI 2.9, rubella AI 2.1). One month later, repeat EMG show widespread sign of acute denervation. As still myopathic MUPs were recorded, a vastus lateralis biopsy was performed, which did not reveal specific abnormalities. Repeat LP confirmed positive MRZ reaction. However, due to the clinical picture and electrophysiology the diagnosis of ALS was favored. One month later, non-invasive ventilation was administered due to progressive dyspnea. Repeat MRI showed dural thickening and enhancement adjacent to the sphenoidal sinuses which showed prominent mucosal swelling. In the following year, the patient was repeatedly admitted for adjustment of NIV parameters. Neurologically a slowly progressive spastic tetraparesis as well as hypaesthesias of left calf and foot were documented. Five years after first admission, the patient reported progressive weakness of both arms, muscles cramps and progressive dysphagia necessitating tube feeding after percutaneous endoscopic gastrostomy. Seven years after initial admittance, gait disturbance had progressed to an extend that he required a wheelchair for longer distances. He reported widespread pain of the right upper and lower extremities. Upon neurological examination, dysarthophonia was documented. Spastic tetraparesis more pronounced on the right was noted associated with reported hypaesthesias of the right arm and leg. Cerebral MRI now showed one subcortical WML and scattered pontine hyperintensities, most likely of vascular origin. Spinal MRI again did not show any spinal cord lesions. Repeat LP now did not show positive OCB, phosphorylated neurofilament heavy chain (pNFH) concentrations in the CSF were below lowest level of detection (cut-off for MND >560 pg/ml). Eight years after initial presentation, the patient reported progressive painful cramps of the esophagus when swallowing. A jackhammer esophagus had been diagnosed and treated with botulinum toxin. Walking distance had shortened to 10 m. Head flexion and extension had become paretic, truncal instability was noted. Repeat NCS now showed demyelinating changes in the arm nerves and EMG widespread chronic and acute neurogenic changes. Due to the unusual course of the disease and paraclinical findings, a second muscle biopsy, which again did not reveal any relevant abnormalities. Thus, the diagnosis of MND was questioned and immunomodulatory therapy with high-dose intravenous immunoglobulins recommended. The patient was lost to follow-up.

**Patient 18: Sjögren’s syndrome, leukocytoclastic vasculitis and slight tetraparesis with predominant arm weakness**

This 70-years-old female was admitted to our department for diagnostic work-up of a progressive slightly asymmetrical weakness of both arms pronounced on the right, which had started two months ago. Retrospectively, she reported that she had experienced some uncommon fatigue of her leg muscles when climbing stairs starting 4 months earlier. An EMG performed prior admission had shown acute neurogenic changes in the right biceps and right tibial anterior muscles. Plasma CK had been found elevated in plasma. Six years earlier, she had developed a dry mouth upon which Sjögren’s syndrome had been diagnosed serologically. Three years later, she had experienced a rash of her legs. This had recurred less than one year later. Cutaneous biopsy had revealed leukocytoclastic vasculitis and had been treated with topical corticosteroids without inducing remission. Diagnostic work-up as in inpatient in the department of dermatology three years prior admission to our department had shown erythematous macules and petechiae as well as hyperpigmentation covering legs and feet. Ophthalmological examination had revealed keratoconjunctivitis sicca. General laboratory examination had shown elevated CK (397 U/l, <155 U/l). Rheumatological serology revealed an increases ANA titer with highly elevated anti-Ro- and anti-La antibodies. Topic clobetasol 17-propionate and compression wrapping had induced remission. A relapse of rash had occurred three months prior admission to our department and had been treated with systemic methylprednisolone followed by oral tapering to 1 mg at admission.

Upon neurological examination, mild paresis of both arms, more pronounced on the right, was noted. In addition, getting up from a squat position was slightly impaired, indicating minimal paresis of the legs. There were no additional abnormalities. Cerebral MRI showed some subcortical WML in line with cerebral microangiopathy. Spinal MRI did not show any spinal cord lesions. NCS showed reduced amplitude in the right peroneal nerve. EMG in two muscles innervated by cervical and lumbal roots showed signs of acute and chronic neurogenic changes. CSF analysis showed normal leukocyte count (0 leukocytes/µl, upper normal limit 4 leukocytes/µl), minimal blood-CSF barrier function (9.5 x 10^-3^, age-dependent upper normal limit 8.7 x 10^-3^). Positive OCB restricted to the CSFG were found, but no evidence of quantitative intrathecal IgG, IgA or IgM synthesis. Measles and rubella AIs were elevated (5.2 and 4.5, respectively), while VZV AI was normal (0.7). CK still was elevated in the range found three years ago (379 U/l) as was blood sedimentation rate (73 mm/h).

Oral methylprednisolone was stopped prior dismissal and the patient was again admitted 4 weeks later for muscle biopsy of the left deltoid muscle. Conventional histopathology examination of the deltoid muscle did not reveal any obvious neuropathic or myopathic changes. However, immunohistology demonstration of endomyseal CD4+ more than CD8+ lymphocytic infiltrates, perivascular CD20+ B cell infiltrates, many MHC-positive muscle fibers as well as complement deposits in some medium-sized vessels. Following biopsy, oral prednisolone 1 mg/kg bodyweight for five days was prescribed followed by 50 mg oral methylprednisolone. Slow tapering was advised. No follow-up data were available.

**Patient 19: Arthralgias and atypical motoneuron disease**

Six weeks before admission to our department, the 50-year-old male had been treated as an in-patient in another hospital due progressive weakness and muscle wasting starting about two months earlier. In addition, he had reported to suffer from arthralgias for years due to psoriasis arthritis. Previously, also diabetic neuropathy had been diagnosed. The neurological examination had revealed distally pronounced symmetrical tetraparesis. He had reported stocking-type hypaesthesias of the legs. Distal pallanaesthesia of both feet had been found. Cerebral MRI had shown three unspecific subcortical white matter lesions, spinal MRI had been normal. NCS revealed decreased amplitudes for sensory nerves, but normal motor NCS. EMG had shown widespread acute and chronic neurogenic changes compatible with motoneuron disease (MND). CSF examination had revealed pleocytosis (21 leukocytes/µl, upper normal limit 4 leukocytes/µl), normal total protein but positive OCB restricted to the CSF. CRP had been slightly (22 mg/L) and ESR highly elevated (38 mm/h, 91 mm/2h). Upon muscle biopsy, myositis had been excluded but neurogenic changes had been found. Rheumatological serology had not shown any abnormalities. He had been treated with high-dose methylprednisolone for 5 days. Arthralgias had been improved as had mobility. Repeat LP 12 days later had shown normal cell count (0 leukocytes/µl).

Upon neurological examination at our department, to which he was admitted for a second opinion, exaggerated deep tendon reflexes were found at the lower extremities. EMG and NCS findings again were compatible with sensory PNP combined with MND. Sensory evoked potential from the right leg and VEP from the right eye showed increased latencies. Repeat CSF showed slight pleocytosis (5 leukocytes/µl), slight blood-CSF barrier dysfunction (Q_Alb_ 8.1 x 10^-3^, age-dependent upper normal limit 7.3 x 10^-3^) and positive OCB restricted to the CSF without evidence for quantitative intrathecal Ig synthesis. Onconeuronal antibodies were negative. CRP was elevated (33 mg/L). As ALS could not been excluded, riluzole was started. Upon examination in our outpatient clinic three months later, the patients reported progressive weakness of the extremities, slurred speech, shortness of breath and daytime sleepiness. Bulbar palsy of the tongue was noted. No mutations in SOD1, VAPB, TARDBP, FUS and C9ORF72 genes were found. The patient was re-admitted to start non-invasive ventilation. CRP still was elevated (66 mg/L). Repeat-CSF showed normal cell count (3/µl), normal Q_Alb_ (6.5 x 10^-3^), positive OCB, a normal measles AI (1.3) in combination elevated rubella (1.6) and VZV AIs (1.9). Upon continuous non-invasive ventilation at night, daytime sleepiness improved, but weakness progressed during follow-up. One year after the first LP, a follow-up LP was performed, which showed normal cell count (2 leukocytes/µl), normal Q_Alb_ (6.7 x 10^-3^) and still positive OCB. Two years after initial LP, the patient only reported a very slow progression of muscle weakness. Upon last follow-up 3.5 years after first hospital admission, the patient reported that muscle weakness had not progressed since the previous examination 1.5 years earlier.

**Patient 20: Patchy sensory loss associated with Crohn’s disease after discontinuation of immunosuppression with azathioprine**

This 55-year-old female was referred to our department for neurological work-up. The patient reported a fluctuating numb feeling of both legs that firstly had appeared eleven years before the admission. Furthermore, the patient had developed unsteady gait and stance. The patient had a high cardiovascular risk profile (insulin-dependent diabetes mellitus, harmful use of alcohol, 20 pack years of tobacco smoking). In addition to having suffered from Crohn’s disease for 25 years, the patient had suffered a tuberculosis 20 years ago with recent reactivation during the year preceding admission. Crohn’s disease had been treated with azathioprine until she had developed an acute pancreatitis three months prior to admission and was thus discontinued.

Upon neurological examination, ankle jerks were absent, no pareses could be found. The patient reported of patchy areas with reduced sense of touch on the lateral side of both lower legs. Cerebral MRI showed mild periventricular, probably microangiopathic WML. Spinal MRI was not performed. Blood tests showed increased γGT (461 U/L, normal <55 U/L) and slightly increased CRP (20 mg/L, normal <5 mg/L). HbA1c was elevated to 11% (normal <6.5%). No deficiency of B vitamins was detectable. NCS showed absent sensory nerve action potentials and reduced amplitudes of motor compound action potentials of the lower limbs. Correspondingly, EMG showed signs of acute and chronic neurogenic changes in the anterior tibial muscle. The findings were interpreted to be consistent with an axonal polyneuropathy. CSF analysis revealed a normal leukocytes count (4 leukocytes/µL, upper normal limit 4/µl leukocytes), slightly perturbed blood-CSF barrier function (Q_Alb_ 8.8 x 10^-3^, age-dependent upper normal limit 7.7 x 10^-3^). Lactate was normal (2.7 mmol/L, normal ≤2.7 mmol/L). Unexpectedly, positive OCBs restricted to the CSF associated with quantitative evidence of intrathecal IgG synthesis (54%) were found. A second CSF analysis performed two days later confirmed OCB restricted to the CSF associated with quantitative intrathecal IgG synthesis of 57%. In addition, measles, rubella as well as VZV AIs were elevated (3.4, 11.5 and 2.1, respectively). Again, cell count (0 leukocytes/µL) was normal and CSF-blood barrier function slightly perturbed (Q_Alb_ 8.9 x 10^-3^). As time of dismissal, it was assumed that the poorly controlled diabetes mellitus was most likely responsible for the neurological deficits. Strict control of blood glucose levels was recommended.

Seven years later, the patient was admitted to our depart of ophthalmology due loss of vision on the right eye. Central retinal artery occlusion was diagnosed. The admission files indicated that relapsing-remitting multiple sclerosis had been diagnosed in the year following the admission to our department. No additional details became available.

**Patient 21: Axonal neuropathy and serology suggestive for Sjögren’s syndrome**

This 76-year-old female was admitted due to a rapid progression of a pre-existing gait disturbance.

Upon admission, the patient’s body temperature was elevated (38.1°C). Neurological examination revealed slight paraparesis. Somatosensory function of touch reportedly was normal. However, sensation for vibration was reduced on the right malleolus (4/8) and lost on the left malleolus. Deep tendon reflexes of both legs were reduced. Cerebral MRI revealed periventricular white matter lesions. Spinal MRI was not performed. NCS demonstrated axonal and sensory neuropathy of both legs. CRP was highly elevated (264 mg/L, normal <5 mg/L). Blood count revealed leukocytosis (20.3 G/L, normal 4.3-10.8 G/L). However, no concurrent infection was identified. Rheumatologic serology showed borderline normal anti-nuclear antibodies (1:100), highly elevated SSA/Ro antibodies (>240 U/ml, normal <7 U/ml), Ro52 antibodies (<240 U/ml, normal 7/ml) as well as Ro60 antibodies (133 U/ml, normal <7 U/ml). SSB/La antibodies were normal. CSF analysis showed normal leukocyte count (1/µl, upper normal limit 4/leukocytes µl), no blood-CSF-barrier dysfunction (Q_Alb_ 5.6 x 10^-3^, normal 9.1 x 10^-3^), no evidence for quantitative intrathecal immunoglobulin synthesis but positive OCB restricted to the CSF. Measles AI was normal (0.8), rubella and VZV AIs were elevated (2.5 and 1.9, respectively). CSF lactate was normal (1.3 mmol/L).

An infection-induced exacerbation of and preexisting gait disturbance due to pre-existing sensory neuropathy was diagnosed, antibiotic therapy initiated, and the patient was transferred to another hospital for additional work-up and continuation of the antibiotic therapy. A re-admission for Schirmer test and lip biopsy was planned. However, the patient was lost to follow-up.

**Patient 22: Hemifacial spasm with inflammatory CSF findings**

This 57-year-old female was admitted for further diagnostic work-up of repeated twitching of her left corner of the mouth starting four months ago. Three months earlier, cranial CT and EEG had been performed in another department of neurology without any abnormalities. LP had shown normal CSF leukocytes count (4 leukocytes/µl, upper normal limit 4 leukocytes/µl) but isolated OCB in the CSF. Ten years ago, she reportedly had suffered from borreliosis with arthralgias and myalgias. She reported to have a hypaesthetic area located at her right groin following an episode of shingles in that area five years ago. She also suffered from persistent foot drop on the right diagnosed as traumatic peroneal nerve palsy.

Upon neurological examination, continuous twitching of left corner of the mouth without facial palsy was noted. Foot as well as toe extension on the left were paretic. There was a hypaesthetic area at her right groin. Video-EEG monitoring with a suspected diagnosis of epilepsia partialis continua did not show any abnormalities. Cerebral MRI only showed a single periventricular white matter lesion, in addition a possible nerve-vessel contact of the vertebral artery and the facial nerve on the right was suggested. A spinal MRI was not performed. Nerve conduction studies demonstrated an axonal lesion of the left facial nerve. Borreliosis serology was negative. Repeat-LP showed normal leukocyte count (0 leukocytes/µl), minimal blood-CSF barrier dysfunction (Q_Alb_ 7.9 x 10^-3^, upper normal limit 7.8 x 10^-3^), no evidence for quantitative intrathecal IgG, IgA or IgM synthesis. OCB restricted to the CSF were positive. Bother measles and rubella AIs were elevated (3.9 and 1.7, respectively). However, VZV AI (0.9) and HSV AI (0.7) were normal.

In summary, the diagnosis of hemifacial spasm was made. As the additional findings suggested a chronic intrathecal inflammatory origin of the facial nerve lesion additional work-up and follow-up in our outpatient clinic was advised. As currently borreliosis serology was negative, the previous diagnosis of borreliosis as cause for the episode of arthralgias and myalgias became questionable. The patient was lost to follow-up.

**Patients rated to suffer from multiple sclerosis rather than a non-MS CAIND**

**Patient 23: Frontal executive dysfunction followed by vertical gaze palsy and progressive spastic paraparesis**

The 52-year-old female was admitted for neurological work-up after psychiatric in-patient treatment. Five months prior admission, she had developed emotional lability and depression. Two months later, she had suddenly developed dizziness, gait instability and fatigue. These problems had persisted after initiation of antidepressant therapy with mirtazapine. In addition, she had started to report forgetfulness and difficulties to concentrate.

Upon admission in our department, neurological examination did not reveal any abnormalities except for slight pallhypaesthesia at both ankles (5/8). Neuropsychological testing revealed prominent executive dysfunction with perseveration and slowed cognitive processing speed. Cerebral MRI showed multiple subcortical and periventricular WML with contrast enhancement in one subcortical lesion. NCS were normal as well tibial nerve SEP. CSF showed normal leukocyte count (1 leukocytes/µl, upper normal limit 4 leukocytes/µl) but 3% plasma cells upon cytological examination. Blood-CSF-barrier function was normal (Q_Alb_ 3.2 x 10^-3^, age-adjusted upper normal <7.5 x 10^-3^). Isolated OCB restricted to the CSF were detection in conjunction with 30% quantitative intrathecal IgG synthesis, no quantitative evidence of intrathecal IgA or IgM synthesis. Lactate was normal (1.8 mmol/L). Measles and VZV AIs were elevated (11.7 and 5.0, respectively). The rubella AI was normal (1.4). Tau protein (216 pg/ml, <300 pg/ml) and Amyloid beta 1-42 (601 pg/ml, >550 pg/ml) were normal. CRP was slightly elevated (6.2 mg/L, <5 mg/L), rheumatological serology showed no abnormalities.

As a chronic inflammatory disease was assumed, high-dose methylprednisolone (1g/day for 5 days) was administered. Upon follow-up four months later, the patient reported that corticosteroids had not led to any changes of her symptoms. Follow-up cranial MRI did not show any new lesions. Spinal MRI did not show any lesions of typical size and shape for demyelination. In the following time, the patient’s symptoms remained largely stable. Next follow-up cranial MRI revealed one additional lesion in the periventricular white matter. Two years after first admission, gait stability had deteriorated. In addition, neurological examination revealed supranuclear vertical gaze palsy. Later, repeated falls ensued. She developed hypophonia and dysphagia as well as slight spastic paresis of the left leg as well as gait ataxia. Normal plasma levels of lyso-SM-509 could exclude Niemann-Pick disease type C. Genetic testing excluded Huntington’s disease. At last follow-up four years after first admission, increased spasticity of the left arm had developed. Deep tendon reflexes of the legs were exaggerated, plantar responses were extensor bilaterally. She was only able to walk with help. Cognitive status hadn’t changed over the last years.

**Patient 24: Probable paraneoplastic optic neuritis associated with renal clear cell carcinoma**

This 50-year-old female was admitted via the department of ophthalmology due to loss of vision of the nasal visual field of her left eye that suddenly had developed two days earlier. Ophthalmological examination had shown reduced visual accuracy of the left eye (0.5) as well as restriction of the nasal visual field and red desaturation of this eye. Prior to these neurological symptoms, a space-occupying lesion of her left kidney had been detected by ultrasound.

Neurological examination confirmed loss of vision in the left nasal field associated with reduced visual accuracy. No other abnormalities were found. Cerebral MRI revealed a left cerebellar ring-enhancing lesion and another pontomesencephalic contrast-enhancing lesion, both without perifocal edema. A non-enhancing WML was found subcortically in the left insular region. Spinal MRI was not performed. Lumbar puncture showed pleocytosis (23 leukocytes/µL, upper normal limit 4 leukocytes/µl). Cytological examination of the CSF showed lymphocytes and monocytes as well as 9% plasma cells. No tumor cells were found. Blood-CSF barrier function was normal (7.2 x 10^-3^, upper normal limit 7.3 x 10^-3^). Lactate was normal (1.3 mmol/L). Oligoclonal IgG restricted to the CSF was positive. Q_IgG_ was elevated with 12% intrathecal IgG synthesis, no evidence of quantitative intrathecal IgA and IgM synthesis was noted. Measles AI was elevated (4.3), while rubella AI was borderline normal (1.4) and VZV AI normal (0.5). Repeat-LP five days later for flow-cytometry and repeat cytology showed 11% B-cells but no monoclonal B-cell population. Now CSF leukocyte count was 17/µL, no tumor cells. Q_Alb_ had decreased to 5.5 x 10^-3^. In turn, quantitative intrathecal IgG synthesis increased to 27% and MRZ reaction had become positive with AIs for measles, rubella and VZV to 5.8, 1.9 and 0.6, respectively. CRP in plasma was slightly increased (28.7 mg/l, upper normal value 6 mg/l). CT of the chest and abdomen was highly suggestive for a renal cell carcinoma of the left kidney with a pulmonary lesion compatible with a metastasis as well as pathological retroperitoneal lymph nodes. Paraneoplastic and aquaporin-4 antibodies were negative. Rheumatologic serology was negative. Monoclonal gammopathy IgG kappa of unknown significance was diagnosed. The patient was referred to department of urology for nephrectomy and periaortal lymphadenectomy. Pathological examination confirmed renal clear cell carcinoma (pT3a, L0, V0, R0, GII). Post-surgery, 100 mg prednisolone was started orally. Corticosteroid treatment was associated with subjective improvement of vision within several days. Upon dismissal, ophthalmological examination could not confirm an increase in visual accuracy, however the visual field restriction appeared improved.

Seven years later, the patient was admitted to our department of surgery for cholecystectomy due to a gallbladder stone that was detected during cancer aftercare. Apparently, no recurrence of the renal cell carcinoma had occurred. Multiple sclerosis was listed among the diagnoses in the discharge report without any additional information available.

**Patient 25: Predominantly axonal peripheral neuropathy with possible central involvement partially responsive to immune adsorption**

The 62-year-old male presented to our department due to progressive weakness of both legs, which had started on the right foot three years ago. When climbing stairs, he would develop muscle pain in his thighs. He had observed muscle twitching in his right thigh. His toes had become numb. Repeatedly, this numbness would be accompanied by burning sensations. He also reported painful cramps of both legs, mostly at night. Ten months ago, an inflammatory neuropathy had been diagnosed and treated with a course of high-dose dexamethasone without any beneficial effect. Later, intravenous immunoglobulins had also failed to induce any improvement of his condition.

Neurological examination upon admission showed marked atrophy of the right thigh and fasciculations in both thighs. There was slight weakness of the right hip and knee flexion and extension as well as of both feet. The right patellar tendon reflex was absent. Both ankle jerk reflexes were present, however more pronounced on the right. Plantar response on the right tended to be extensor. Hypaesthesia for touch was noted on both feet as well as in the right calf. Cerebral MRI showed multiple periventricular as well as subcortical WML and one juxtacortical WML, spinal MRI showed no lesions. NCS revealed asymmetrical mostly axonal neuropathy, while tibial SEP showed marked slowing. EMG of muscles of both arms and the left leg as well as paraspinal muscles was normal, while signs of acute and chronic denervation were found in the right lateral vastus muscle. CSF showed slight pleocytosis (5 leukocytes/µl, upper normal limit 4 leukocytes/µl) and moderate blood-CSF barrier dysfunction (Q_Alb_ 14.5 x 10^-3^, age-dependent upper normal limit 8.1 x 10^-3^). OCB were positive without any evidence for quantitative intrathecal Ig synthesis. Measles, rubella, VZV as well as HSV AIs were elevated (6.1, 4.5, 5.1 and 6.1, respectively). Rheumatologic serology and onconeuronal antibodies were negative.

A chronic inflammatory neuropathy with possible CNS involvement was suspected. Oral prednisolone was started, followed by slow tapering. Again, no treatment response was noted upon follow-up. Screening for an underlying neoplasm led to the detection of a prostate adenocarcinoma several months later, which was treated by surgery and radiotherapy. However, prostatectomy was not followed by stabilization of the neuropathy. Fourteen months after initial admission, paraparesis had progressed to a degree that the patient required a walker. After immune adsorption, the patient slightly improved. In the following years, 10 cycles of immune adsorption were performed, which at first were associated in a temporary improvement for several months that became less with each cycle. Finally, rituximab was administered seven years after first admission. No information with regard to the effect of this treatment is available.

**Appendix 2: Tabular details of the non-MS PSIIR-positive patients**

| **No.** | **Gender** | **Age** | **Symptoms preceding admission and during hospital stay** | **Clinical findings** |
| --- | --- | --- | --- | --- |
| 1 | f | 75 | two generalized tonic–clonic seizures after colon cleansing prior colonoscopy | transient impairment of consciousness, no other focal neurological deficits |
| 2 | m | 52 | generalized tonic–clonic seizure for 3 min 12 month after hanta virus infection | no focal neurological deficit, tongue bite left |
| 3 | m | 81 | 2 years of progressive cognitive impairment | cognitive impairment |
| 4 | f | 66 | episodes of confusion for 4 months. 1 day of aphasia, apraxia and right arm weakness | aphasia and central paresis of the right arm (due to MCA ischemia), deficits of orientation in time, mental processing speed, and impairment of executive functions (probably not caused by MCA ischemia) |
| 5 | m | 60 | initial episode of vertigo, 8 months of progressive weakness first the left and then the right arm associated with muscle wasting and twitching. Episode of vertigo 6 years earlier | Atrophy, distally pronounced paresis of the left arm with exaggerated reflexes, paresis of right finger extensor, generalized fasciculations |
| 6 | m | 82 | dysphagia and slurred speech starting one year ago | pseudobulbar palsy, hyperreflexia upper limbs |
| 7 | f | 86 | unconsciousness and left myocloni followed by two bilateral tonic-clonic seizures at day of levofloxacin due to urinary tract infection | initially impaired consciousness, later awake and no focal neurological deficit |
| 8 | m | 68 | chronic headache now exacerbated. Fluctuating consciousness, severe depression. History of epilepsy, sensorimotor neuropathy as well as reportedly 30 episodes of meningitis | psychomotor slowing, depression, gaze-evoked nystagmus, dysarthria, atactic gait |
| 9 | m | 51 | reportedly fluctuating paresthesias of hands and feet, sleep attacks, amnestic episodes, intermittent chest pain, lumbalgia | no neurological deficits, probably somatoform disorder |
| 10 | m | 60 | 13 months of personality changes, cognitive impairment, slurred speech and left-sided spastic hemiparesis | right-sided facial paresis, dysarthophonia, left-sided spastic paresis, executive dysfunction, impairment of mental processing speed and verbal episodic memory |
| 11 | m | 54 | 5 days of persistent headache | no focal neurological deficits |
| 12 | m | 63 | 14 days of persistent headache, recurrent fever (up to 39°C) followed by bilateral hearing loss and left hypoglossal nerve palsy | no focal neurological deficits at admission, later bilateral hypoacusis loss and left hypoglossal nerve palsy |
| 13 | f | 66 | 2 weeks of headache and impaired vision acuity on the left eye | visual accuracy of the left eye reduced (0.6), distally pronounced pallhypesthesia, imbalance in Romberg’s test |
| 14 | m | 80 | acute dizziness and nausea following otitis media on the right, preexisting gait disturbance due to peripheral neuropathy | nystagmus to the left upon Dix-Hallpike maneuver, spontaneous nystagmus to the right, absent ancle jerks, stocking type hypesthesia’s |
| 15 | f | 78 | 6 months of slowly progressive weakness of the right little finger with extension deficit, pre-existing Raynaud phenomenon and dysphagia due to esophageal dysmotility | finger extension paresis (dig V>dig. II-IV), dig V abduction paresis. Asymmetric ankle jerks (left > right). Slight pallhypesthesia, minor disequilibrium upon tandem walking |
| 16 | m | 57 | slowly progressive gait disturbance, numbness and painful dysesthesias of the legs | spastic tetraparesis, thermhypesthesia right leg, pallhypesthesia both legs, extensor plantar response bilaterally |
| 17 | m | 49 | 1 year of progressive paraparesis starting on the left side, radiating pain in the medial ventral right thigh, generalized muscle twitching and cramps | li>re spastic paraparesis, muscle atrophy left thenar, the right thigh and both calves |
| 18 | f | 69 | 2 months of progressive asymmetrical arm weakness, 4 months of minimal weakness of the legs | mild paresis of both arms more pronounced on the right, minimal paresis of the legs |
| 19 | m | 50 | >2 months of progressive weakness and muscle wasting, known psoriasis arthritis. Diagnosis of diabetic neuropathy | symmetrical tetraparesis, exaggerated deep tendon reflexes of the legs, stocking-type hypesthesia’s, pallanaesthesia of both feet |
| 20 | f | 44 | 11years of fluctuating hypesthesia of the lower limbs, unsteady gait and stance | absent ankle jerks, hypesthesia of the lateral aspects of both lower legs |
| 21 | f | 76 | rapid progression of pre-existing gait disorder | slight flaccid paraparesis, hyporeflexia and bilateral malleolar pallhypesthesia, fever (38.1 °C) |
| 22 | f | 57 | 4 months of left facial muscle twitching of fluctuation intensity, history of borreliosis with arthralgias and myalgias, as well as shingles with hypesthesia left groin and foot drop following reportedly traumatic peroneal nerve palsy | continuous twitching of left corner of the mouth without facial palsy, left foot and toe extension paresis, hypaesthetic area at her right groin |
| 23 | f | 52 | 5 months of emotional lability, depression; 2 months dizziness, gait instability and fatigue, disturbance of memory | executive dysfunction, perseveration, slowed cognitive processing speed, distal pallhypesthesia |
| 24 | f | 50 | 2 days of loss of vision in the nasal visual field of the left eye | restriction of the left nasal visual field and decreased visual acuity of the left eye (0.5) |
| 25 | m | 62 | 3 years of progressive paraparesis. Exercise-induced myalgias and cramps, numbs toes, painful cramps in both legs | right>left paraparesis, right triceps surae atrophy, fasciculation of both thighs, right patellar tendon reflex was absent, ankle jerks r>l, extensor plantar response r, distal hypesthesia r>l |

| **No.** | **Course** | **Symptoms at progression** | **Imaging** | **CSF findings** |
| --- | --- | --- | --- | --- |
| 1 | acute | - | cerebral MRI: mild periventricular + juxtacortical WML, moderate global atrophy; spinal MRI: n.d. | 1 cell/µl, Qalb normal, OCB+, AIs: M 3.0, R 1.1, Z 1.5 H neg. |
| 2 | acute | - | cerebral MRI: normal; spinal MRI: n.d. | 2 cells/µl, Qalb normal, OCB+, AIs: M 1.7, R 1.2, Z 3.7 |
| 3 | chronically progressive | - | cerebral MRI: r. cerebellar subacute ischemia, periventricular WML; spinal MRI: n.d. | 0 cells/µl, Qalb normal, OCB+, AIs: M 0.9, R 1.9, Z 1.9, H 3.4, Tau protein increased (824 pg/ml), Abeta1-42 decreased (527 pg/ml) |
| 4 | chronic with exacerbation | no neurological symptoms documented 11 years later | cerebral MRI: subacute l. parietotemporal MCA ischemia, periventricular + juxtacortical WML, mesiotemporal atrophy, spinal MRI: n.d. | 1 cells/µl, Qalb normal, OCB+, 11% IIS IgG, AIs: M 4.8, R 2.2, Z 2.6, H 0.9 |
| 5 | chronically progressive | progressive tetraparesis, respiratory failure, bulbar + pseudobulbar palsy | cerebral MRI: periventricular + juxtacortical WML, spinal MRI: normal | 2 cells/µl, Qalb/lim 1.4, OCB+, AIs: M 2.4, R 0.9, Z 2.2, H neg. |
| 6 | chronically progressive | - | cerebral: few periventricular WML, spinal: normal | 1 cell/µl, Qalb normal, OCB+, AIs: M 1.4, R 1.5, Z 1.0, H 1.7, pNFH 5619 pg/ml (> 560 pg/ml) |
| 7 | acute | - | cerebral MRI: moderate microangiopathy with periventricular + subcortical WML (report only); spinal MRI: n.d. | 1 cells/µl, Qalb normal, OCB+, AIs: M 1.7, R 1.7, Z 1.6, H 0.8 |
| 8 | chronic with exacerbation | episodes of disorientation and reduced consciousness | cerebral MRI: periventricular + juxtacortical WMLs; spinal MRI n.d. | 18 cells/µl, Qalb normal, OCB+, IIS IgM 37%, AIs: M 1.0, R 4.9, Z 1.9, H 3.0 |
| 9 | chronic with exacerbation | - | cerebral MRI: few subcortical WML; spinal MRI: n.d. | 0 cells/µl, Qalb/lim 1.5, OCB+, AIs: M 2.3, R 2.6 Z 3.4, hypocretin normal |
| 10 | chronically progressive | - | cerebral: periventricular + subcortical WML, spinal: normal | 1 cell/µl, Qalb borderline normal, OCB+, AIs: M 2.1, R 2.1, Z 1.5; month 7: 0 cells/µl, IIS neg., OCB+, AIs: M 2.0, R 2.0, Z 1.1 |
| 11 | subacute | - | cerebral MRI: no WML, dural thickening + contrast enhancement; spinal MRI: n.d. | 6 cells/µl, Qalb normal, 19% IgG IIS, OCB+, AIs: M 2.4, R 1.2, Z 4.9 |
| 12 | chronic with exacerbation | hoarseness, dysphagia, l. hearing loss, l. facial numbness | cerebral MRI: no WML, frontal sinusitis; spinal MRI: n.d. | day 14: 49 cells/µl, Qalb/lim 1.3, OCB-, day 23: 35 cells/µl, Qalb/lim 1.2, OCB+, day 34: 17 cells/µl, OCB+, AIs: M 0.7, R 4.0, Z 1.8, H 1.2, year 14: 2 cells/µl, OCB+, AIs: M 0.7, R 1.0, Z 1.0, H 2.1 |
| 13 | subacute | - | cerebral: Multiple subcortical + juxtacortical WML, spinal: n.d. | 1 cell/µl, Qalb normal, OCB+, IIS IgG 17%, AIs: M 1.6, R 2.6, Z 4.5, H 1.8 |
| 14 | chronic with exacerbation | 2 years later: probable seizure with disorientation, dysarthria and right-sided hemiparesis; 9 years later vasculitic AION r. eye, giant cell arteritis | cerebral MRI: report only, opacification of the mastoid air cells on the r. 2 years later: cerebral MRI: periventricular WML, spinal MRI: no lesions | 1 cell/µl, Qalb normal, OCB+, AIs: M 1.6, R 1.5, Z 1.8 |
| 15 | chronically progressive | - | cerebral MRI: periventricular WMLs, MRI cervical + upper thoracic spinal cord: normal | 3 cells/µl, Qalb normal, OCB+. IgG IIS 41%, IgA IS 47%, AIs: M 4.5, R 1.9, Z 6.0, H 1.9 |
| 16 | chronically progressive | urinary urge, progressive spastic tetraparesis | cerebral MRI: few subcortical WML, spinal MRI: normal. | 10 cells/µl, Qalb borderline normal, OCB+, IIS IgG 39%, M 3.6, R 4.9, Z 1.0, H 0.6, month 7: 1 cell/µl, IIS neg., OCB+, AIs: M 2.5, R 5.7, Z 1.0 |
| 17 | chronically progressive | l. oculomotor + r. hypoglossal palsy, tetraparesis, respiratory failure, dysphagia,  jackhammer esophagus, neuropathic pain | cerebral MRI: normal, spinal MRI: normal. Cerebral MRI at year 5: 1 subcortical WML, scattered pontine hyperintensities, spinal MRI: normal | 0 cells/µl, Qalb normal, OCB+, AIs: M 1.9, R 1.5, Z 1.2,  8 yrs later OCB- |
| 18 | chronically progressive | - | cerebral: MRI subcortical WML, spinal MRI: normal | 0 cells/µl, Qalb/lim 1.1, OCB+, AIs: M 5.2, R 4.5, Z 0.7 |
| 19 | chronically progressive,  later stable | progressive tetraparesis, bulbar palsy + dysarthria | cerebral MRI: subcortical WML, spinal MRI: normal | initial CSF 21 cells/µl, OCB+, later 3 cells/µl, Qalb normal, OCB+, AIs: M 1.3, R 1.6, Z 1.9 |
| 20 | chronic with exacerbation | central retinal artery occlusion l. eye 7 years later, MS listed among diagnoses | cerebral MRI: mild periventricular WML, spinal MRI: n.d. | 0 cells/µl, Qalb/lim 1.1, OCB+, IIS IgG 57%, AIs: M 3.4, R 11.5, Z 2.1 |
| 21 | chronic with exacerbation | - | cerebral MRI: periventricular WMLs; spinal MRI: n.d. | 1 cell/µl, Qalb normal, OCB+, AIs: M 0.8, R 2.5, Z 1.9 |
| 22 | chronic with exacerbation | - | cerebral: single periventricular WML, spinal: n.d. | 0 cells/µl, Qalb borderline normal, OCB+, AIs: M 3.9, R 1.7, Z 0.8, H 0.7 |
| 23 | chronically progressive | supranuclear gaze palsy, dysphagia, dysarthophonia, spastic tetraparesis | cerebral MRI: multiple subcortical + periventricular WML, enhancement in 1 subcortical lesion; spinal MRI: normal | 1 cell/µl, Qalb normal, OCB+, 30% IIS IgG, AIs: M 11.7, R 1.4, Z 5.0 |
| 24 | acute | unknown | cerebral: left cerebellar ring-enhancing lesion, pontomesencephalic contrast-enhancing lesion, subcortical WML, spinal: n.d.; CT-thorax/abdomen: l. renal cell carcinoma | day 2: 23 cells/µl, Qalb normal, OCB+, IIS IgG 12%, AIs: M 4.3, R 1.4, V 0.5; day 7: 17 cells/µl, Qalb normal, OCB+, IIS IgG 27%, AIs: M 5.8, R 1.9, Z 0-6 |
| 25 | chronically progressive | progressive paraparesis | cerebral MRI: periventricular + subcortical WML, 1 juxtacortical WML; spinal MRI: normal | 5 cells/µl, Qalb/lim 1.8, OCB+, AIs: M 6.1, R 4.5, Z 5.1, H 6.0 |

| **No.** | **Electrophysiology /Ultrasound** | **Laboratory findings** | **Immunosuppressive treatment** | **Response** | **Follow-up** | **MS upon  follow-up** | **Cancer** | **Other autoimmune disease** |
| --- | --- | --- | --- | --- | --- | --- | --- | --- |
| **1** | - | hyponatremia (126mmol/l), hypochloridemia (95 mmol/l) | none | - | - | - | none | none |
| **2** | EEG: intermittent r. frontotemporal slowing | CK 3583 U/l | none | - | - | - | none | none |
| **3** | - | onconeur. abs neg., rheumatologic serol. neg. | none | - | - | - | none | none |
| **4** | EEG: r. frontotemporal slowing, ultrasound: l. ICA stenosis | Pos. anti-histone and ds-DNA abs | none | - | 11 yrs | no | None* | none |
| **5** | EMG: generalized acute + chronic neurogenic changes | rheumatol. serol. neg., CRP 8.0 mg/l, leukocytes 11.2 G/l | none | - | 2 yrs | no | none | none |
| **6** | EMG: acute + chronic neurogenic changes m. genioglossus, not in the arm and leg muscles | - | none | - | -- | - | none | none |
| **7** | - | CRP 32 mg/l, leukocytes 20 G/l | none | - | - | - | none | none |
| **8** | - | CRP 157 mg/L, leukocytosis (13.5 G/l), urinary tract infection | none | - | - | - | none | none |
| **9** | EEG: normal, NCS: normal, quantitative sensoric testing: normal, Schellong test: normal | onconeur. abs neg., rheumatol. serol. neg. | none | - | - | - | none | none |
| **10** | SEP: prolonged latencies, TMS: normal CMCT, NCS: normal, EMG month 13: acute denervation lateral vastus m. only | onconeur. abs neg., muscle biopsy: unspecific changes | none | - | 13 months | no | none | atrophic gastritis |
| **11** | - | CRP 145 mg/l | none | - | - | - | none | none |
| **12** | - | - | none | - | 14 yrs | no | none | none |
| **13** | NCS: axonal r. peroneal n. lesion, EMG ant. tib. m.: acute + chronic neurogenic changes, tibial SEP: normal, VEP: normal, retinal fluoresceine angiography: normal | rheumatol. serol. neg., CRP normal, blood sedimentation rate 30 mm/h | 100 mg prednisolone | fast/ complete | - | - | none | none |
| **14** |  |  | 3 days 250 mg prednisolone. followed by oral tapering | fast/ complete | 9 yrs | no | none | giant cell arteritis 9 yrs later |
| **15** | NCS: normal, EMG: normal | onconeur. abs neg., ANA >1:2560, centromere abs >240 U/ml; | planned, but unknown if started | - | - | - | none | CREST syndrome |
| **16** | TMS: increased CMCT, NCS: normal. | Rheumatol. serol. neg., Aq-4 abs neg. | HDMP followed by oral prednisolone | partial/ temporary | 4.5 yrs | no | none | none |
| **17** | NCS: axonal sensory neuropathy, later demyelination. EMG: acute+chronic neurogenic changes l. abductor pollicis m. only, later some myopathic MUPs | onconeur. abs neg., Aq-4 abs neg., muscle biopsy: no specific abnormalities | planned in year 8, but not done | - | 8 yrs | no | thyroid | none |
| **18** | EMG: acute+chronic neurogenic changes, NCS: axonal lesion r. peroneal n. | ANA, anti-Ro and -La antibodies pos., CK elevated (379 U/l), muscle biopsy: compatible with myositis | oral prednisolone with tapering | unknown | 4 weeks | no | none | Sjögren's syndrome, leukocytoclastic vasculitis |
| **19** | NCS: axonal sensory neuropathy, EMG: acute + chronic neurogenic changes compatible with LMND | CRP 33 mg/l, BSR highly elevated (38 mm/h, 91 mm/2h), onconeur. abs neg., rheumatol. serol. neg., muscle biopsy: neurogenic changes | HDMP | partial/ temporary | 3.5 yrs | no | none | Psoriatic arthritis |
| **20** | NCS legs: SNAP -, CMAP reduced amplitudes, EMG ant. tib. Muscle: acute + chronic neurogenic changes | yGT 462 U/l, CRP 20 mg/l, | none | - | 7 yrs | yes | none | Crohn's disease |
| **21** | NCS: axonal sensory neuropathy | CRP 264 mg/l, leukocytes 20.3 G/l, SSA/Ro, Ro52 and Ro60 abs pos. | none | - | - | - | none | Sjögren's syndrome (laboratory) |
| **22** | NCS: axonal lesion facial n., video EEG monitoring: normal | borreliosis serol.: neg. | none | - | - | - | none | none |
| **23** | Tibial SEP: normal, NCS: normal | CRP 6.2 mmol/l, rheumatol. serol. neg., lyso-SM-509 normal,  HD excluded | HDMP | none | 4 yrs | no | none | none |
| **24** | - | CRP 28.7 mg/l, onconeur. + Aq-4 abs neg., rheumatol. serol. neg., MGUS IgG kappa | 100 mg prednisolone | minimal | 7 years | yes | renal | none |
| **25** | NCS: asym. axonal neuropathy, tibial SEP: delayed bilaterally, EMG: M. vastus med. r.: acute + chronic neurogenic changes | onconeur. abs neg., rheumatol. serol. neg. | oral dexamethasone, oral prednisolone. 90mg, immune adsorption | partial (immune adsorption) | 7 yrs | no | prostate | none |

*Renal carcinoma 11 yrs later. Abbreviations: AI = antibody index, AION = anterior ischemic optic neuropathy, ANA = antinuclear antibodies, Aq-4 = aquaporin-4, BSR = blood sedimentation rate, CK = creatine kinase, Cl. = cluster, CMCT = central motor conduction time, CREST = calcinosis-Raynaud phenomenon-esophageal involvement-sclerodactyly-telangiectasia syndrome, CSF = cerebrospinal fluid, CT = computed tomography, EEG = electroencephalography, EMG = electromyography, f = female, H = Herpes simplex, HD = Huntington’s disease, HDMP = high-dose methylprednisolone, ICA = internal carotid artery, IIS = intrathecal immunoglobulin synthesis, l. = left, LMND = lower motoneuron disease, LP = lumbar puncture, m = male, M = measles, MCA = middle cerebral artery, MGUS = monoclonal gammopathy of undetermined significance, MRI = magnetic resonance imaging, MS = multiple sclerosis, MUPs = motor unit potential, NCS = nerve conduction study, n.d. = not done, , neg. = negative, OCB = oligoclonal bands onconeur. = onconeural, pNFH = phosphorylated neurofilament heavy protein, PSIIR = polyspecific intrathecal immune response, Qalb = CSF/serum albumin ratio, Qalb/lim = age-dependent upper Q_Alb_ level of normal, r. = right, rheumatol. = rheumatologic, R = Rubella, SEP = somatosensory evoked potentials, TMS = transcranial magnetic stimulation, VEP = visual evoked potentials, WML = white matter lesions, yrs = years, Z = Varicella zoster
